# Robust Skin-Conformal Nano-Electrodes for Sustainable Health and Performance Monitoring

**DOI:** 10.1021/acsnano.5c08540

**Published:** 2025-08-12

**Authors:** Jinyoung Kim, Sehyun Park, Jisoo Jeon, Dong-hee Kang, Gwendolyn M. Bryan, Timothy J. Broderick, Morley Stone, Vladimir V. Tsukruk

**Affiliations:** † School of Materials Science and Engineering, 1372 Georgia Institute of Technology, Atlanta, Georgia 30332, United States; ‡ 115599 Institute for Human and Machine Cognition, Pensacola, Florida 32502, United States; § Department of Intelligent Systems and Robotics, University of West Florida, Pensacola, Florida 32514, United States

**Keywords:** flexible nanoscale films, skin-conformal nano-electrodes, wearable sensors, health and performance monitoring, low-motion artifact, underwater monitoring

## Abstract

Wearable electrodes with high conformability to the skin allow for a second-skin-like wearing experience and record high-quality electrophysiological signals over extended time in challenging environments. However, current research on skin-conformal electrodes faces limitations due to excessive motion artifacts under real-life external conditions. Here, we report a nanoscale skin-conformal electrode that enables continuous resilient electrophysiological signal monitoring with highly suppressed noise, low-motion artifacts, and high water-resilience, all unachievable with commercial gel electrodes. In particular, achieving a conformal skin–electrode interface provides mechanical and electrical stability under repeated dynamic stress (5000 times). The 300 nm nano-electrodes with dual hydrophilicity integrate a hydrophilic nanoscale 2D MXene conductor and a hydrophobic cross-linked parylene layer, ensuring highly conformal contact and long-term stable physical adherence to skin. This ultrathin design facilitates high physical adhesion and low skin interfacial impedance for continuous, reliable monitoring of electrocardiogram (ECG), and electromyogram (EMG) signals with a greatly increased signal-to-noise ratio. As a proof of concept, we successfully recorded high-quality ECG signals, allowing for the analysis of heart rate across diverse in-field testing. We further demonstrated concurrent EMG and ECG monitoring during treadmill walking, achieving stable, long-term signal acquisition, particularly in monitoring demanding human activity.

## Graphical abstract

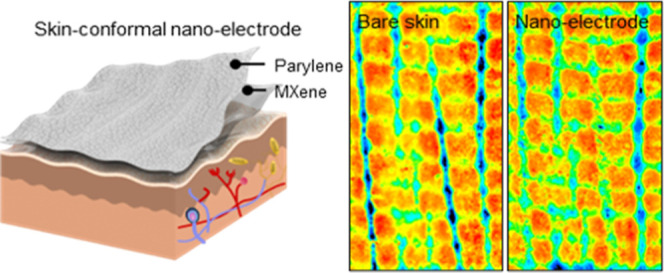

## 1 Introduction

Skin-mounted sensory electronics requires durable wearable sensors that adhere directly to the skin for various applications such as healthcare monitoring, fitness tracking, and human–machine interaction.^1–3^ Notably, epidermal electrodes placed on the skin enable the accurate extraction of electrophysiological signals such as electrocardiogram (ECG),^4^ electromyogram (EMG),^5^ electrooculogram,^6,7^ and electroencephalogram.^8,9^ These electrodes offer continuous monitoring of biopotentials, aiding in the diagnosis and treatment of cardiac, neurological, and muscular disorders as well as assessing skin impedance and hydration levels. Specifically, accurate detection, resilience to motion artifacts, and long-term environmental/water stability are great concerns for real-time health monitoring.^10^

Conventional electrophysiological electrodes, such as gel-type and dry contact electrodes, face several critical limitations, including high motion artifacts, high skin interfacial impedance, low signal-to-noise ratio (SNR), and short-term usage under harsh environmental conditions. Specifically, gel-type electrodes are short-lived and prone to motion artifacts because the Ag/AgCl gel electrolyte is vulnerable to environmental factors and sweat absorption.^11^ These factors lead to poor unstable contact to the skin and decreased signal quality over a long time of use. In addition, dry contact electrodes such as thin metal,^12–15^ conductive polymer,^16–18^ carbon-based^19,20^ and composite-based^21^ films rely on direct contact with the skin surface. However, this contact is affected by air gaps, the loss of contact, and tribological charging, which severely compromise electrophysiological signals, particularly in dynamic environments. There has been growing interest in improved electrophysiological electrodes such as ultrathin electrodes,^6,22^ e-tattoos,^19,23^ and smart adhesives^24,25^ for direct mounting on the skin.

It has been suggested that ultrathin flexible electrodes provide close contact on dynamic curvilinear surfaces due to low bending stiffness for firmly attaching to the skin.^26^ However, achieving a seamless, highly adherent, and void-free contact with skin remains challenging, as air gaps tend to form at the skin–electrode interface. This issue is more pronounced in micrometer-thick electrodes, which exhibit greater mismatch in interfacial mechanics, leading to motion artifacts during intense shearing and bending at the electrode–skin interface.

In particular, the control of hydrophilicity on the surface is relatively unexplored, though refining this aspect could prevent water penetration at the skin–electrode interface. Tuning surface hydrophilicity can significantly improve electrical contact and adhesion in wet physiological environments, highlighting the critical role of interfacial wetting engineering for water management in bioelectronic systems.^27^ Such strategies can further provide enhanced mechanical/electrical stability and reliable electrophysiological signal acquisition for extended times even in dynamic and water-rich environments.

Herein, we introduce a skin-conformal nano-electrode with hydrophilic and hydrophobic sides comprising a highly conductive layer of a 2D MXene phase (Ti_3_C_2_T_*x*_),^28–31^ a poly-l-lysine (PLL) adhesive layer, and an ultrathin cross-linked CVD-fabricated parylene nanofilm (Figure 1). The transfer process onto human skin, using water-assisted capillary-driven flow, reinforced the high conformability of the skin–electrode interface without air voids, tight physical contact, and high skin adherence. This ultrathin hydrophobic–hydrophilic trilayered design facilitates the resistance to low-motion artifacts and continuous monitoring of ECG and EMG signals under challenging external conditions and long sustainable performance in air and underwater unachievable by current thick gel and dry electrodes.

**Figure 1:**
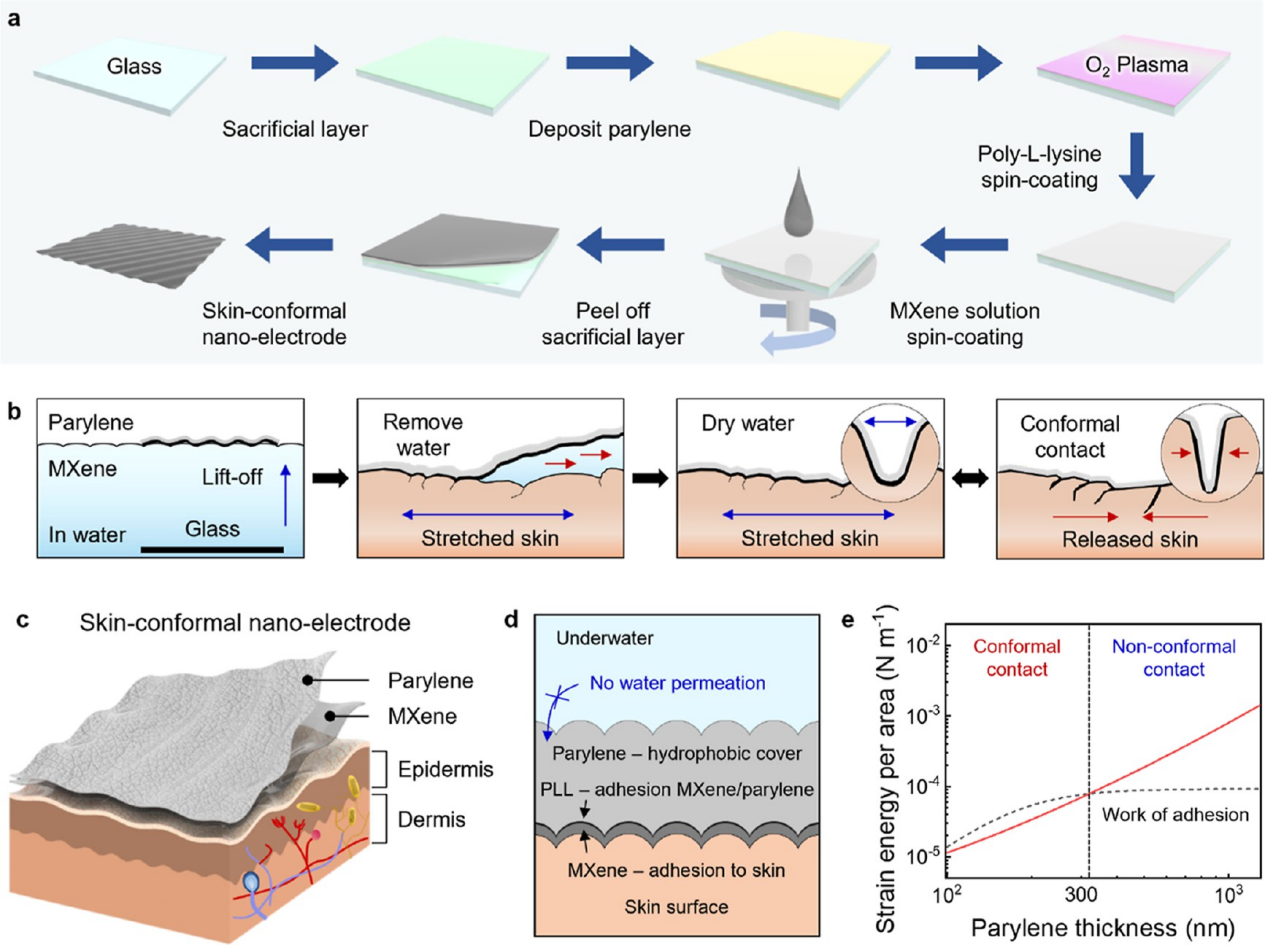
Skin-conformal nano-electrodes for electrophysiological signal detections. (a) Schematic illustrating the fabrication process of the skin-conformal nano-electrode. (i) Coating a sacrificial layer on a cleaned glass substrate. (ii) CVD-based preparation of a parylene substrate with the desired thickness. (iii) Treating the substrate with O_2_ plasma and spin-coating with (iv) PLL and (v) MXene. (vi) Peeling the as-prepared MXene/parylene electrode from the glass substrate by dissolving the sacrificial layer in DI water. (b) Schematic illustrating the transfer process of nano-electrodes onto human skin for conformal contact. (i) The MXene/parylene film floats on the water surface before gently being placed on the stretched skin surface. (ii) Any residual water between the MXene/parylene film and the skin surface is removed during drying. The skin-mounted nano-electrodes enable stable conductive performance when the skin is (iii) stretched and (iv) released. (c) Schematic of skin-conformal nano-electrodes. (d) Schematic of water-resistant skin-conformal nano-electrodes based on the dual hydrophilicity. (e) Determination of parylene thickness required for conformal contact as a function of nano-electrode thickness and the work of adhesion of human skin.

In comparison to commercial gel electrodes, the skin-conformal nano-electrodes demonstrated exceptional ECG and EMG signal quality, characterized by manifold (up to 3 times) increase in signal-to-noise ratio, dramatically reduced noise, and showed low-motion artifacts and sustainability. For proof of concept, we used our skin-conformal nano-electrodes to monitor ECG signals, enabling the determination of heart rate (HR) and HR variability (HRV) both in extreme (sauna and swimming pool) environments unachievable with existing sensors. Finally, we successfully demonstrated the concurrent monitoring of EMG and ECG signals for high-quality muscle activity during a walking cycle and long-term (up to 30 h) stability.

## 2 Results

### 2.1 Fabrication and Device Design

We fabricated the skin-conformal nano-electrodes using a spin-coating and sacrificial layer-etching process in Figure 1a (see the detailed fabrication process in the Experimental Section). Briefly, we first fabricated a sacrificial layer on a cleaned glass substrate, followed by parylene chemical vapor deposition and spin-coating with the PLL adhesive molecular layer for attaching a nanoscale conducting layer of staggered 2D flakes of MXenes.

To achieve enhanced skin interfacial contact and long-term stability, the rolling transfer process of skin-conformal nano-electrodes onto human skin was implemented (Figure 1b) (see the detailed transfer process in Figure S1, and Video S1). We placed the as-fabricated MXene/parylene electrodes in deionized (DI) water to dissolve the sacrificial layer and release it in the free-standing state. Different hydrophilicities of the parylene (air) and MXene (water) interfaces make the MXene/parylene electrodes float on the water without forming any crumples. After removing the residual water and allowing it to dry completely, the MXene/parylene electrodes can be conformally attached to the prestretched skin, leading to high skin–electrode contact, stability, and durability when the skin is released.

The MXene layer acts as a conductive layer helping to perform not only high-level electrophysiological signal detection but also strong hydrophilic and polar interactions with skin and the PLL layer. This materials design yields a hydrophilic surface and high adhesion between negatively charged MXene nanosheets and the positively charged amine functional groups of PLL (zeta potential of −22.6 and +26.3 mV, respectively (Figure S2)). In addition, a flexible hydrophobic parylene layer provides the integrity of the sensory film and chemical stability. The hydrophilic side faces the skin surface, while the hydrophobic surface faces outward as shown in Figure 1c. This dual hydrophilicity design of the skin-conformal nano-electrodes prevents direct water penetration at the skin–electrode interface, enabling water-resistant behavior (Figure 1d).

To determine the optimal electrode thickness (known as critical thickness) for conformal contact with the skin, we calculated the bending-induced strain energy per area and work of adhesion using an analytical model for interfacial mechanics (see Figure 1e and details on calculations in Supporting Information Note 1).^32,33^ Firm conformal contact is achieved when the work of adhesion of the electrode exceeds the bending-induced strain energy. Here, the electrode in this model consists of two layers: an MXene layer (layer 1) and a parylene layer (layer 2). Based on the optimized thickness of the MXene layer as 50 nm, we analyzed the critical thickness of the parylene layer, where conformal contact occurs. The bending-induced strain energy (*U*_bending‑strain_) per area of the electrode can be evaluated from^34^

1 $$\frac{U_{bending‐strain}}{A}=\frac{E_{eff}\cdot t}{24R^{2}}$$

2 $$E_{eff}=\sum_{i=1}^{N}\frac{E_{i}\cdot t_{i}}{t_{total}}$$

where *A* is an area of the electrode, *E*_eff_ is the effective elastic modulus of the electrode, and *R* is the radius of curvature. In addition, *E*_eff_ is calculated as a composite of *N* layers, *t*_total_ is the total thickness of the electrode, *E*_*i*_ is the Young’s modulus of the *i*th layer, and *t*_*i*_ is the thickness of the *i*th layer. *R* was considered to be approximately 1 mm for skin.^35^

In addition, for the work of adhesion (γ) on human skin by the function of the parylene thickness, the interfacial mechanics are given as

3 $$\gamma =\frac{\left(\frac{\pi^{4}\cdot EI\cdot h^{2}}{\lambda_{rough}^{4}}+\frac{{\pi \cdot E}_{skin}{\left(h-h_{rough}\right)}^{2}}{16\lambda_{rough}}\right)}{\frac{{\pi^{2}\cdot h}^{2}}{4\lambda_{rough}^{2}}+1}$$

where EI is the effective bending stiffness, *h* is the maximum deflection of the electrode, *E*_skin_ is the skin modulus, *h*_rough_ is the skin roughness amplitude, and λ_rough_ is the skin wavelength.^26^

Then, we plot the bending-induced strain energy per area (represented by the red line) and the work of adhesion (represented by the dotted line) in Figure 1e. The intersection of these boundaries occurs at about 300 nm, providing theoretical limits for conformal nano-electrode behavior. Therefore, fabrication design and following measurements were conducted using 300 nm-thick films (denoted below as nano-electrodes).

### 2.2 Structural Analysis of Conformal Coatings

Next, we investigated the conformability of the nano-electrodes on a skin replica and compared it with 1- and 3 μm-thick electrodes by using a profilometry height profile analysis with a three-dimensional (3D) laser microscopy (Figure 2a). The profile image of the nano-electrode (right) exhibited highly conformal contact, closely following the bare skin (left) (Figure 2b). Moreover, along the dashed black line and gray area, we analyzed the height profile and 3D areal surface roughness analysis, respectively.

**Figure 2:**
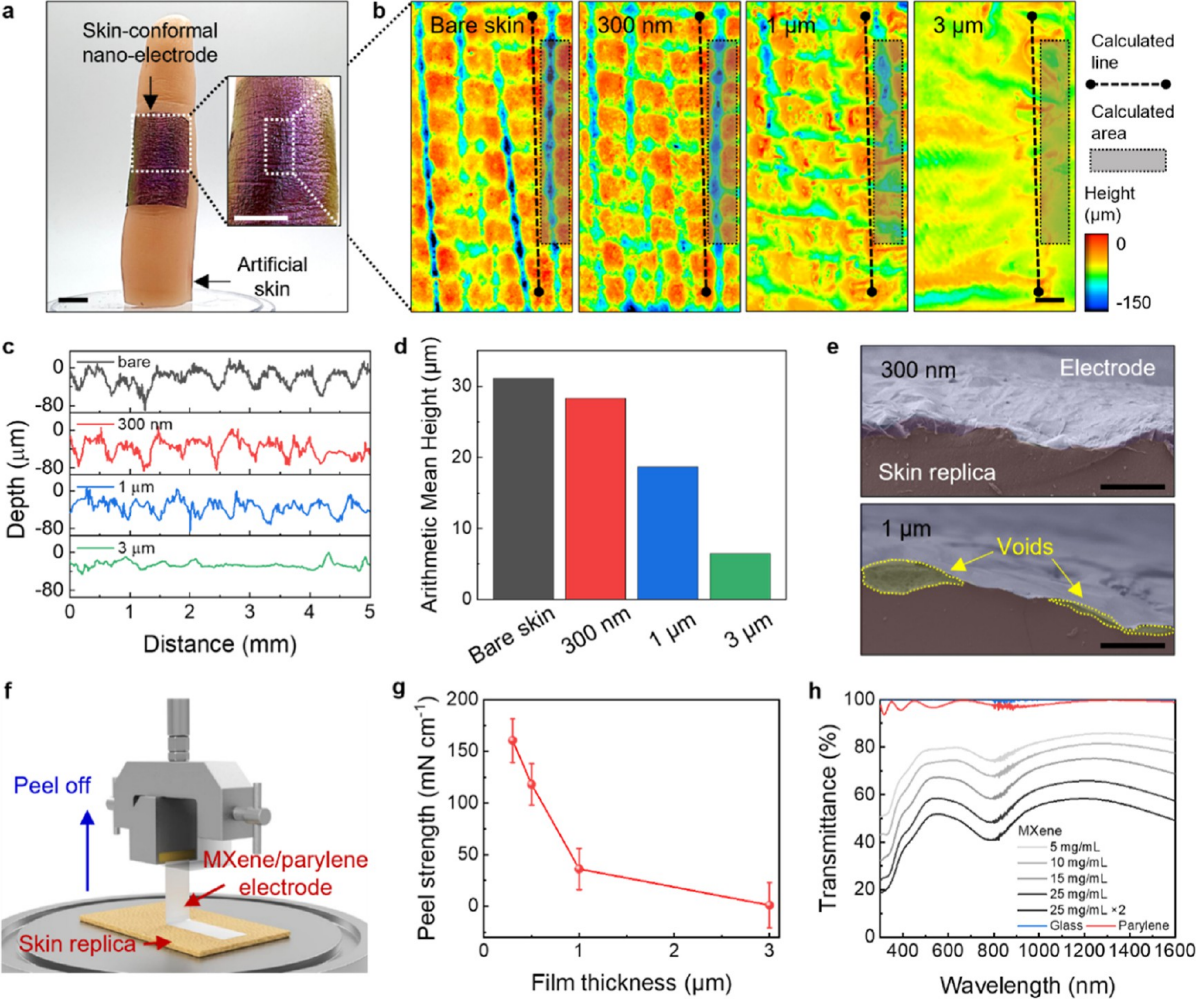
Skin-conformal nano-electrodes onto artificial skin. (a) Photographs of a skin-conformal nano-electrode adhesion onto the artificial skin (scale bar, 2 cm). (b) Profilometry height profiles with different parylene thicknesses (bare skin, 300 nm-, 1 μm-, and 3 μm-parylenes) (scale bar, 0.5 mm). The roughness analysis of (c) linear depth (line: 5 mm) and (d) 3D areal surface (area: 0.5 mm × 3.5 mm) with different thicknesses (bare skin (black), 300 nm- (red), 1 μm- (blue), and 3 μm- (green) parylenes). (e) Cross-sectional SEM images with different electrode thicknesses (300 nm- (top) and 1 μm- (bottom) thick) (scale bar, 100 μm). (f) Schematic of the peeling strength measurement with the same area (1.5 cm × 4.0 cm). (g) Peel strength test of nano-electrodes with different thicknesses delaminated from the artificial skin (peeling rate of 50 mm/min). (h) Optical transmittance of a skin-conformal nano-electrode depending on different MXene concentrations (5–25 mg/mL).

Indeed, the morphology of the skin replica covered with the nano-electrode is similar to that of the skin itself, while a notable decrease in conformal contact occurred in 1 and 3 μm-thick electrodes, leading to reduced adaptation to the skin textures in Figure 2c. We further analyzed the 3D areal surface roughness using an arithmetic mean height (Sa) parameter, as shown in Figure 2d. The Sa values for bare skin and MXene/parylene electrodes with thicknesses of 300 nm, 1 μm, and 3 μm showed 31.1, 28.4, 18.7, and 6.5 μm, respectively. These analyses suggested that the nano-electrode exhibited superior conformability, even covering the deep valleys of the microrough fingerprint texture. Furthermore, the superior conformability of the 300 nm nano-electrodes was confirmed with SEM whereas the 1 and 3 μm-thick electrodes allow for microscopic air pockets (Figures 2e and S3).

To further quantify the skin–electrode adhesion, we conducted peel-off tests on four different electrodes (0.3, 0.5, 1, and 3 μm-thick) on commercial rubber artificial skin (Figure 2f). We observed that the peel strength, calculated as force per unit width of the film, increased significantly, by 2 orders of magnitude with decreasing parylene thickness (Figure 2g). This dependence further confirms the critical role of the increasing contact area between the thinner electrodes and the skin. In Figure 2h, we observed a broad range of optical transmittance (*T* of 51.9–79.3% at 550 nm and 52.8–80.3% at 940 nm) depending on MXene concentrations (within 5–25 mg/mL) (Figure S4). These optical properties suggest potential for integration with multifunctional optical sensing applications such as photoplethysmography (Figure S5).

### 2.3 Nano-Electrodes as Skin-Located Sensors

To investigate nano-electrodes on the human skin, we transferred them on the back of the hand (Figure 3a). The enlarged image exhibited high conformality to the skin but also some adaptability on hair (dashed blue line; Figure 3b). We conducted adhesion tests on the skin with varying hair densities. The results showed that while adhesion modestly (around 30%) decreased as hair density increased, the nano-electrode still maintained high adherence to the skin (Figure S6). By comparison, a 100 nm-thick parylene electrode on the same skin surface can be easily torn especially on the highly strained valley of a fingerprint as shown in Figure S7. In contrast, skin-conformal nano-electrodes of 300 nm thick are extremely stable at common large skin deformations such as spreading, pinching, and twisting, which facilitates stable signal collection and low-motion artifacts as discussed below (Figure 3c).

**Figure 3:**
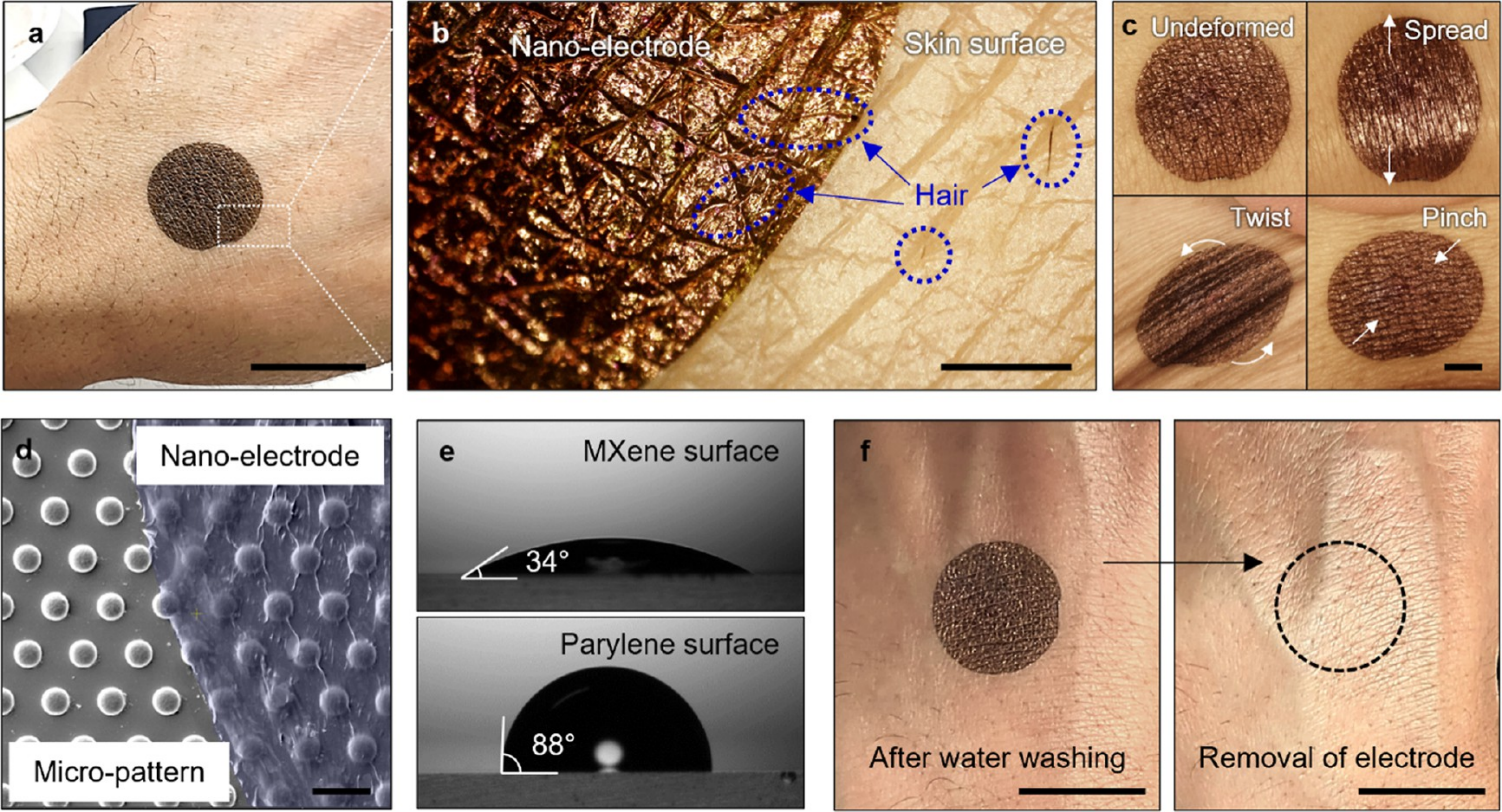
Ultrathin nano-electrodes under different conditions. (a) Photographs of the skin-conformal nano-electrodes onto the human skin (scale bar, 2 cm) and (b) enlarged view of the boundary between human skin and nano-electrodes (scale bar, 50 μm). (c) Photographs of nano-electrodes subjected to undeformed, spread, pinch, and torsion (clockwise, scale bar, 500 μm). (d) SEM image of the electrodes the dome-like patterned array (15 μm diameter and 20 μm pitch) (scale bar, 20 μm). (e) Contact angles of water on the MXene (top) and the parylene surfaces (bottom). (f) Photographs of after cold water (left) and after removal of the skin-conformal nano-electrode with soapy water (right) (scale bar, 2 cm).

Independent testing of an electrode assembly on dome-like patterned polydimethylsiloxane substrates imitating characteristic topographical skin dimensions showed that the nano-electrode completely covered all features (Figure 3d). In contrast, thicker electrodes (0.5 and 1 μm-thick parylene) are suspended over surface features (Figure S8), thus confirming that reaching the critical 300 nm-parylene thickness predicted by theory is essential for full conformal coatings of microscopic topographical features. This conformal coating efficiently eliminates air pockets and increases the total contact area, both of which are critical for electrode integration for enhanced sensing performance, as will be discussed below.

Moreover, the conductive MXene surface showed good wettability with a water contact angle of 34°, which should assist in electrode spreading and facilitate self-adhesion between the electrodes and the skin surface (Figure 3e). This low contact angle is similar to that of cleaned skin (typically 50°–80°).^36^ On the other hand, the outer parylene surface can repel the water droplet as indicated by a higher water contact angle of 88° (Video S2), common for hydrophobic surfaces and oily skin (typically 90–100°), causing water to bead up rather than spread. This unique design of dual wettability allows for strong physical adherence of ultrathin nano-electrodes with dual hydrophobicity.

Meanwhile, the bending stiffness (or flexural rigidity) of the nano-electrode (2.1 × 10^–12^ N·m) is significantly lower than that of human skin (3.8 × 10^–5^ N·m) that facilitates conformal deformation of nano-electrodes at stiffer substrates with larger radii of curvature during deformations (Supporting Information Note 2). The flexibility and low stiffness of the nano-electrodes allow for conformal contact to the skin’s natural topography, particularly in dynamic environments, thus minimizing mechanical mismatch between the electrode and skin.

Such a combination of maximized contact area and matching wettability facilitates strong capillary facilitated physical adhesion of the nano-electrodes to the skin. Indeed, these electrodes sustain strong water flow and rubbing in water (Figure 3f, left*,* and Video S3). Moreover, numerous skin deformations (>6000 times) and sweating during approximately 5 km of running do not compromise nano-electrode attachment (Figure S9). However, the nano-electrodes can be easily removed by handwashing with soap, which delaminates the film by penetrating surfactants (Video S4). Furthermore, physically driven adhesion without the need for additional adhesive tapes prevents excessive skin irritation after electrode washing out (Figure 3f).

Furthermore, we evaluated the breathability of the nano-electrode by measuring its water vapor transmission rate (WVTR) (see Figure S10).^37^ The results show that the nano-electrode exhibits a WVTR of 3.3 mg cm^–2^ h^–1^, which is comparable to common medical dressings (3.1 mg cm^–2^ h^–1^, Tegaderm, 3M Medical),^38^ enabling effective moisture exchange while maintaining strong skin adhesion. This level of breathability helps minimize sweat accumulation beneath the electrode during prolonged wear, thereby reducing and ensuring user comfort. To further evaluate the safety of prolonged skin contact, we conducted cell viability tests, which revealed that after 1, 3, and 6 days of incubation, cell viability remained above 94%, confirming minimal cytotoxicity of the electrodes (see preliminary results in Figure S11).

### 2.4 Electrical Characteristics of the Nano-Electrodes

High conformability at the skin–electrode interface ensures the efficient transfer of electrophysiological signals to the electrode, resulting in greatly increased signal amplitude, minimizing noise and signal attenuation, all critical enhancements not achievable with traditional electrodes (Figure 4). For electrical characterizations of nano-electrodes, we placed three types of electrodes such as nano-, gel, and 1 μm-thick parylene (denoted as thick) electrodes on a volunteer’s forearm.

**Figure 4:**
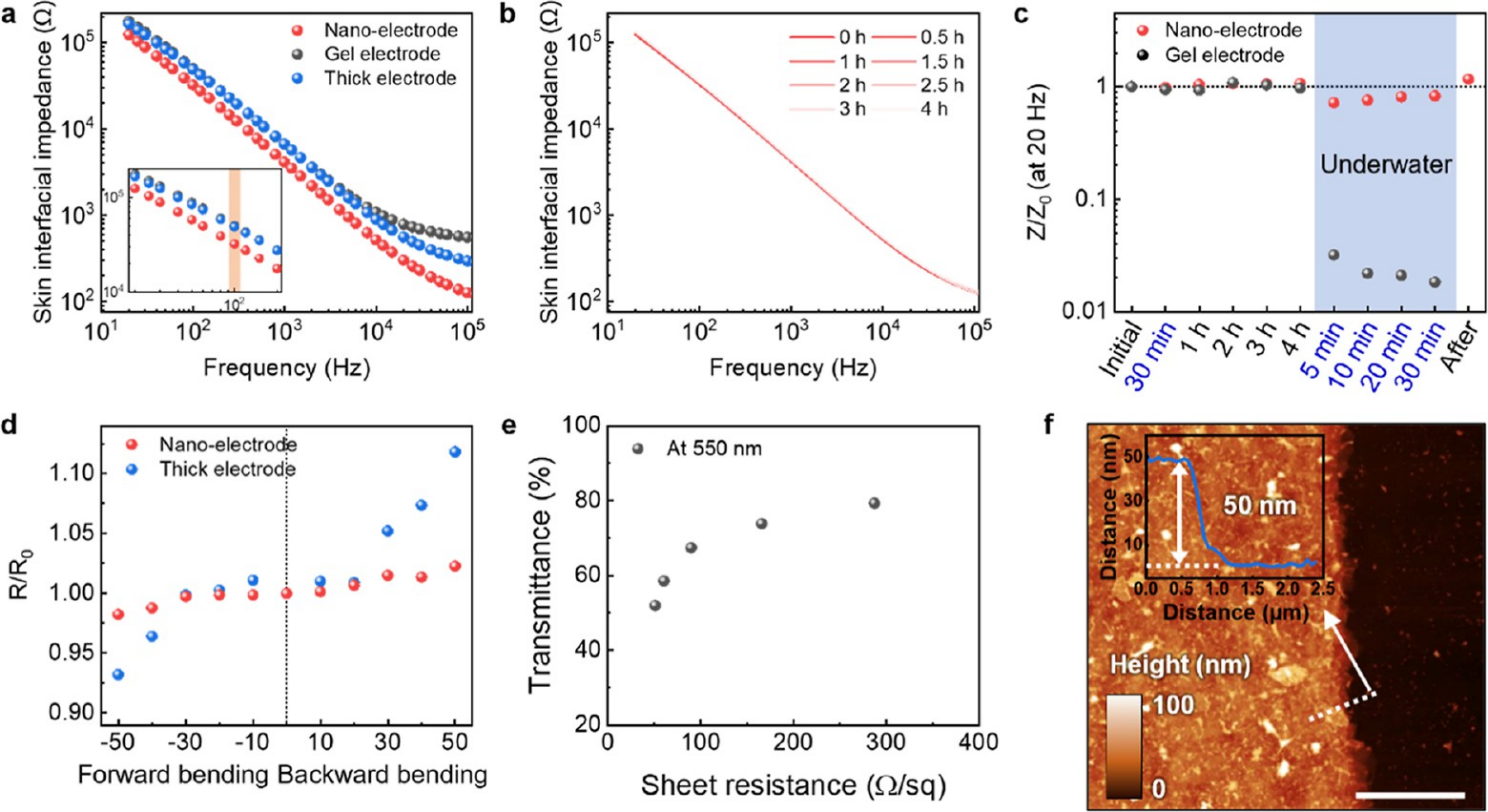
Analysis of the skin–electrode interface. (a) Interfacial impedance of nano- (red), gel (black), and thick (blue) electrodes. The inset shows zoom-in data around 100 Hz. The distance between electrodes was 5 cm, and the contact area of all types of electrodes was around 400 mm^2^ per electrode. (b) Long-term (4 h) impedance stability of the skin-conformal nano-electrodes and (c) corresponding and underwater (30 min) impedances of nano- (red), gel (black) electrodes at 20 Hz. (d) Relative resistance changes of nano- (red) and thick (blue) electrodes as a function of forward and backward bending. (e) Sheet resistance to transmittance at 550 nm (red) for MXene suspensions with different MXene concentrations. (f) AFM analysis for the thickness of the MXene layer from scratch profiling (inset) (scale bar, 5 μm).

First, we observed that the nano-electrode showed a much lower skin interfacial impedance (within 20 Hz to 100 kHz) than that of gel and thick electrodes (Figure 4a). At 100 Hz, the impedance of the nano-electrode (32.3 kΩ) was much lower (about 35%) than that of the gel (50.2 kΩ) and thick (49.0 kΩ) electrodes (Figure 4a, inset). It is noteworthy that the low impedance of the nano-electrodes is due to the skin–electrode contact instead of the conductivity of the electrodes (the sheet resistance of nano- and thick electrodes was 50 Ω sq^–1^). In addition, due to the high conformability, skin-conformal nano-electrodes exhibited long-term impedance stability over a period of 4 h (Figure 4b).

We further evaluated the underwater electrical properties of the skin-conformal nano-electrodes (Figure 4c). The nano- and gel electrodes were immersed in the water following long-term measurements, and the skin interfacial impedance was measured for 30 min as shown in Figure S12. The impedance at 20 Hz of gel electrodes significantly decreased with increased immersion time, with the relative impedance dropping to 0.018 after 30 min of immersion. The reduction in impedance was attributed to water absorption and gel swelling.

After underwater analysis, the gel electrodes lost adhesion to the skin, preventing any further impedance measurements and highlighting a significant limitation of gel electrodes in water-exposed environments. In contrast, the skin-conformal nano-electrodes maintained stable impedance both in air and underwater, particularly following underwater measurements, indicating that electrophysiological signal acquisition is less affected by external environmental conditions. This is attributed to high conformability at the skin–electrode interface and the control of the dual hydrophobicity with low wettability of the parylene and high wettability of the MXene side.

To evaluate the electrical properties of nano- and thick electrodes in response to bending, we attached them to the inner wrist with Ni/Cu tape connected to the sides of the electrode (Figure S13). As observed, the relative resistance changes of the nano-electrode were only ±2% for forward/backward bending (Figure 4d). In contrast, the thick electrode exhibited relative resistance changes of −7% for forward bending and 12% for backward bending.

Furthermore, we assessed the nano-electrode’s electrical durability under repeated 5000 bending cycles on artificial skin, observing high stability and only about 1% relative resistance alternation over long cycling (Figure S14). Moreover, the height profile analysis also confirmed that the nano-electrode maintained firm conformal contact even after 1000 bending cycles, with no significant changes in surface topography after repeated dynamic stresses (Figure S15).

Finally, in addition, to evaluate electrical stability under tensile stress, the nano-electrode was transferred onto a line-patterned silicone elastomer substrate as a deformable model substrate (Ecoflex 00–30, line width: ∼450 μm) while prestretched by 30% in order to evaluate conformal adhesion and structural integrity over the patterned surface (Figure S16). After nano-electrode deposition, we measured relative resistance changes under tensile strain and observed exceptional stability, with negligible variation even at high 30% strain and after 5000 stretching cycles.

Next, we suggest that the conductive layer with low sheet resistance (50.9–287.3 Ω sq^–1^) allows for reliable electrophysiological signals and reduced noise interference during both static and dynamic conditions and maintains a certain level of optical transmittance (with a transmittance of 53–79% at 550 nm) as shown in Figure 4e. Additionally, the nano-electrodes exhibited long-term stability with a negligible resistance change over 10 days (Figure S17).

Overall, the high electrical stability of the nano-electrode, combined with its strong skin–electrode adhesion, enables low-motion artifact and long-term, high-fidelity electrophysiological signal acquisition due to the ultrathin (50 nm) MXene layer (Figure 4f).

Next, we explore if skin-conformal nano-electrodes can be configured into the curvilinear surfaces of the skin while maintaining high capacitance and low impedance, allowing for reliable detections such as ECG and EMG signals.

### 2.5 ECG Analysis for Low-Motion Artifacts under Variable Conditions

In the three-electrode system employed in this study, electrodes were placed on the wrists of the right (RA) and left arms (LA) and on the back of the right-hand as the ground electrode (Figure 5a). ECG signals are composed of several peaks, collectively forming the PQRST waveform, which represents one complete heartbeat cycle, including the P wave, the QRS complex, and the T wave.^39^ As known, each component corresponds to different phases of heart activity, such as atrial and ventricular depolarization and repolarization, for identifying cardiovascular conditions.

**Figure 5:**
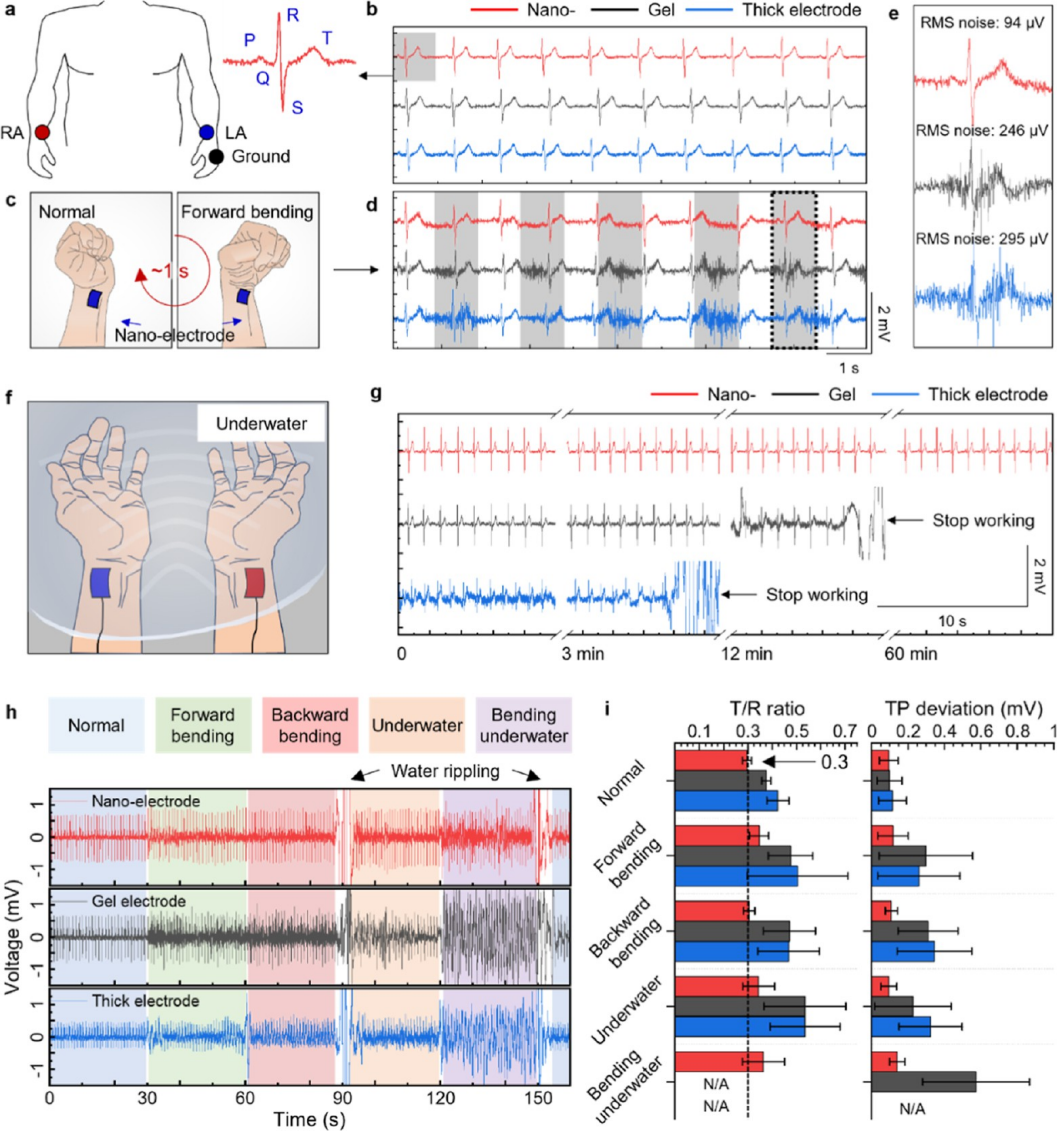
ECG analysis of skin-conformal nano-electrodes in various conditions. (a) Schematic of the ECG measurement setup, in which two electrodes were attached to the inner wrists of the right and left arms, and the other one was attached on the back of the left-hand as the ground electrode. (b) The ECG signals measured by nano- (red), gel (black), and thick (blue) electrodes at the rest condition. (c) Schematics of repetitive normal (left) and forward bending (right) motions with the skin-conformal nano-electrodes attached to the wrist (scale bar, 4 cm). (d) The ECG signals measured by nano- (red), gel (black), and thick (blue) electrodes during repetitive bending motions. (e) Enlarged single ECG signals of nano- (red), gel (black), and thick (blue) electrodes during repetitive bending motions. (f) Schematic of underwater ECG monitoring with skin-conformal nano-electrodes. (g) Long-term underwater ECG monitoring of nano- (red), gel (black), and thick (blue) electrodes. (h) Continuous ECG monitoring of nano- (red), gel (black), and thick (blue) electrodes during activities such as at rest (blue), forward bending (green), backward bending (red), underwater (orange), and forward bending underwater (purple). (i) The analysis of the T/R ratio (left) and TP deviation (right) of nano- (red), gel (black), and thick (blue) electrodes during rest, forward bending, backward, and forward bending underwater.

As shown in Figures 5b and S18, the ECG signals of the nano-electrodes demonstrated higher performance compared to gel and thick electrodes. The nano-electrodes exhibited a superior SNR (35.1 dB), peak-to-peak voltage (1.40 mV), and RMS noise (32 μV), characterized by up to 30% increase in SNR, up to 25% increase in peak-to-peak voltage, and reduced noise by 30–60% compared to the gel and thick electrodes. In addition, the time-dependent voltage signals and corresponding short-time Fourier transform spectrogram allowed for better localization of transient events and the detection of subtle changes in frequency components over short time intervals, identifying irregularities in arrhythmia or ischemic events.

ECG signals were detected during wrist movements, as shown in Figure 5c. We repeatedly performed a normal state and forward bending movement at 1 s intervals. To minimize motion artifacts at the interface between the nano-electrode and the interconnected electrical lead, we developed a device incorporating a mechanical gradient design (Figure S19). Figure 5d shows the ECG signals of the nano-electrode recorded with consistent and clear PQRST peaks under a normal state and forward bending (indicated by the gray blocks), indicating high-fidelity ECG signals with low-motion artifacts.

However, the ECG signals of the gel and thick electrodes made it hard to distinguish PQRST peaks. The RMS noise of the nano-electrode during motions showed a relatively low change (94 μV), whereas the gel and thick electrodes exhibit a much higher RMS noise, which corresponds to the noticeable noise increase (Figure 5e). The increased noise level of the gel and thick electrodes is attributed to shearing at the gel–skin interface (Figure S20). To test the underwater stability further, we attached electrodes to the same location and then completely immersed them in water for 60 min while exercising different activities (Figure 5f).

As observed, the common gel and thick electrodes were detached from the skin almost immediately, within a few minutes, completely destroying the measuring routine. In contrast, the ECG signals of the nano-electrode were consistent and clear PQRST peaks were observed at the longest testing times within an hour (Figure 5g). We suggest that the conformal hydrophobic parylene layer prevents water penetration at the skin–electrode interface, enabling sustainable ECG signal monitoring with low-motion artifacts.

### 2.6 ECG Analysis during Intense Physical Activities

To elucidate the quality of continuous monitoring of ECG signals during wrist movement both in air and underwater (in the order of normal, forward, and backward bending, underwater, and forward bending underwater), we measured the ECG signals for comparison (Figure 5h, the enlarged ECG signals are shown in Figure S21). The applied repeated physical activities generally introduced motion artifact ECG signals, particularly in gel and thick electrodes. Nonetheless, the recorded ECG signals using the nano-electrode demonstrated superior performance, exhibiting reduced noise and consistent signal quality under all tested conditions.

In Figure 5i, we introduce two parameters such as T/R ratio and TP deviation to analyze ECG signals for assessing the heart’s electrical activity and providing insights into various cardiac conditions. First, the T/R ratio is the ratio of the amplitude of the T wave to the amplitude of the R wave. The T and R waves represent ventricular repolarization and depolarization, respectively. It is typically considered to be around 0.30 ± 0.12 in good-quality ECG signals. Accurate monitoring of the T/R ratio is crucial, as an abnormal value (0.18 ± 0.16) may indicate certain cardiac conditions, including ventricular fibrillation.^40^ The nano-electrode maintained a T/R ratio close to 0.3 across all physical activities. However, the T/R ratios for the gel and thick electrodes were higher than the ideal range. Notably, the recorded ECG signals using these electrodes during underwater bending were not instructive.

Furthermore, we analyzed a TP deviation, which is the deviation of the baseline between T and P waves in the ECG signal.^11^ More consistent TP deviations indicate a more reliable ECG signal acquisition. In a normal state, all of the electrodes exhibited similar TP deviations. While the nano-electrodes maintained stable TP deviation values (0.95–1.41 mV) even under bending and underwater conditions, the gel and thick electrodes exhibited significant increases in instabilities, leading to unreliable ECG signals due to poor skin–electrode contact and water absorption/penetration. It is noteworthy that the enhanced stability and reliability of nano-electrodes ensure reduced motion artifacts and effective water-resistant signal monitoring.

### 2.7 EMG Monitoring under Different Conditions

For monitoring EMG signals from the brachioradialis muscle, we mounted the electrophysiological electrodes on the forearm and the back of the hand as the ground electrode (Figure 6a). We conducted an object-grasping experiment using balls with different resistances (in increasing order: stress ball, tennis ball, and baseball). Figure 6b confirms that a higher modulus ball required a greater force to grasp. The SNR of the nano-electrode showed high signal intensity for different stress motions, characterized by a significant (up to 3 times) increase compared to conventional gel and thick electrodes (Figure 6c, Supporting Information Note 3).

**Figure 6:**
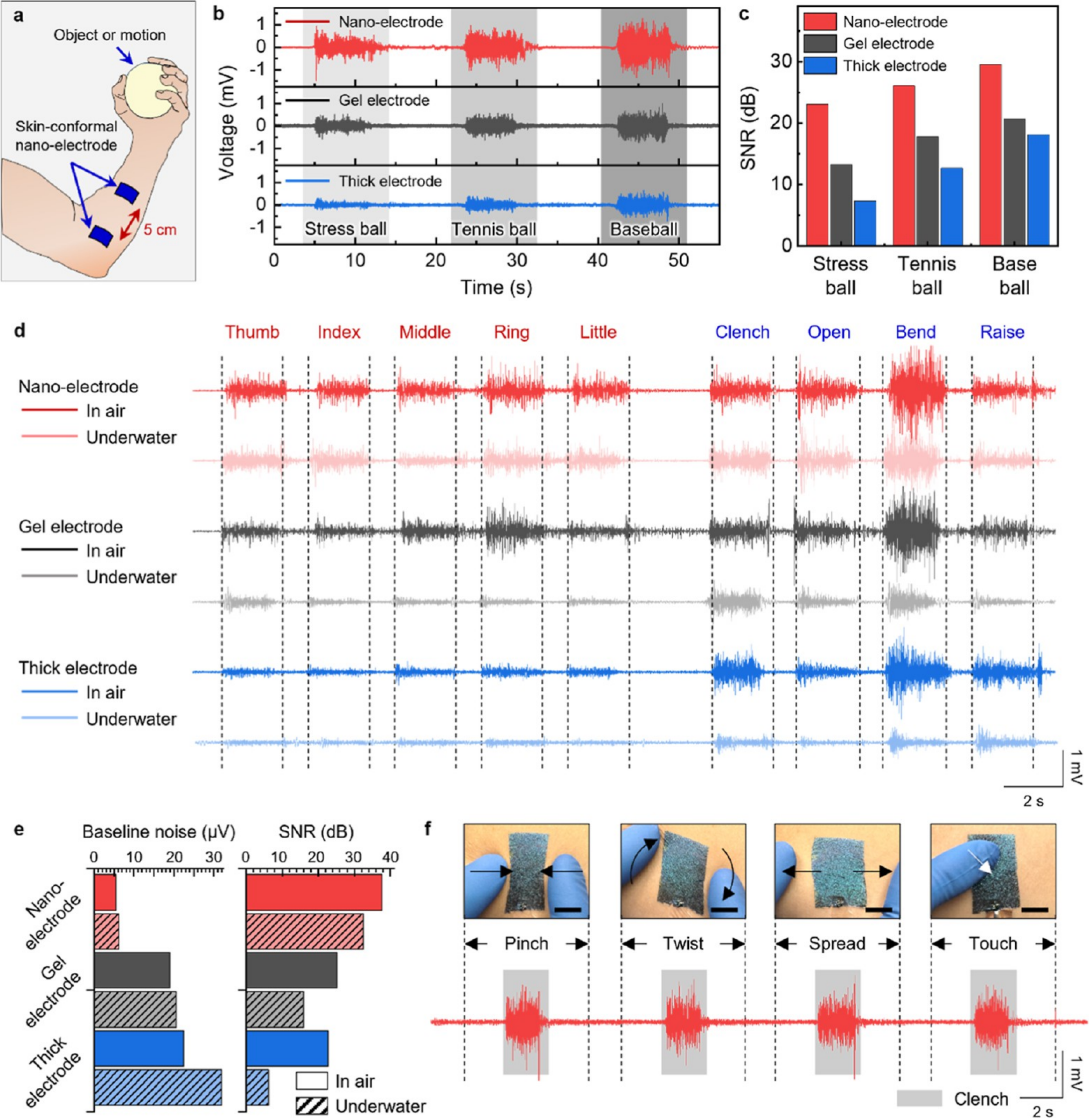
EMG analysis of skin-conformal nano-electrodes in various conditions. (a) Schematic of an EMG measurement setup in which two electrodes were attached to the left forearm and the other one was attached on the back of the right-hand as the ground electrode. (b) The EMG signals are measured by nano- (red), gel (black), and thick (blue) electrodes while gripping the stress ball, tennis ball, and baseball. (c) The SNR analysis of nano- (red), gel (black), and thick (blue) electrodes while gripping the stress ball, tennis ball, and baseball. (d) Continuous EMG monitoring of nano- (red), gel (black), and thick (blue) electrodes in the air and underwater during finger and motion movements. (e) The baseline noise (left) and SNR (right) analysis of bending motion EMG signals in air and underwater (diagonal stripe). (f) Consistent EMG signals under various deformations (pinch, torsion, spread, and touch) (scale bar, 1 cm).

Next, we measured EMG signals of the finger (thumb, index, middle, ring, little) and hand movements (clench, open, bend, and raise) both in air and underwater conditions at 2 s intervals (Figure 6d). To verify the water absorption/penetration to electrodes underwater, the EMG measurement was conducted after immersing the electrodes underwater for 5 min. The results confirmed the water-resistant EMG signals of the nano-electrodes, as they maintained stable and reliable EMG signal acquisition even after immersion. This demonstrated the enhanced durability and functionality of the electrodes in both air and underwater conditions. While the current single-channel configuration limits the discriminability of fine finger movements due to overlapping muscle activations, this limitation is not intrinsic. Rather, it highlights the potential for enhanced gesture recognition when integrated into multielectrode systems, providing more sophisticated and robust EMG-based human–computer interfaces.

We further analyzed the baseline RMS value and SNR of the bending motion (Figure 6e). It is important that the EMG signals of the nano-electrodes demonstrated high consistency both in air and underwater (Videos S5 and S6). Specifically, the baseline RMS value for the nano-electrodes (5.5 and 6.1 μV) was lower by 71–76% in air and 70–81% in underwater conditions. Furthermore, the SNR for the nano-electrodes (37.7 and 32.5 dB) was 1.5–1.7 times higher in air and 1.5–5.2 times higher in underwater conditions compared to gel and thick electrodes. The greater performance differences, beyond the 35% lower skin interfacial impedance, stem from the nano-electrode’s skin conformability and adhesion in dynamic conditions, ensuring stable impedance, reduced motion artifacts, and enhanced signal quality.

Finally, our skin-conformal nano-electrodes demonstrated consistent EMG signals during clench motions under various deformations including pinch, twist, spread, and touch, ensuring accurate and reliable EMG signal acquisition and practical applications in dynamic environments (Figure 6f). This consistency is attributed to baseline stability under the deformations (Video S7). Therefore, our skin-conformal nano-electrodes provide reliable monitoring of muscle activity in diverse environments, critical for versatile wearable technology.

### 2.8 HR Monitoring in Different Testing Environments

The skin-conformal nano-electrodes were used to monitor continuous ECG signals at extreme conditions such as a sauna and a pool (Figure 7a). The ECG data were collected over 25 min, encompassing normal conditions, in a sauna, at rest, and in a pool (see protocol in Supporting Information Note 4). This comprehensive testing sequence was designed to assess the skin’s ability to reliably capture ECG signals.

**Figure 7:**
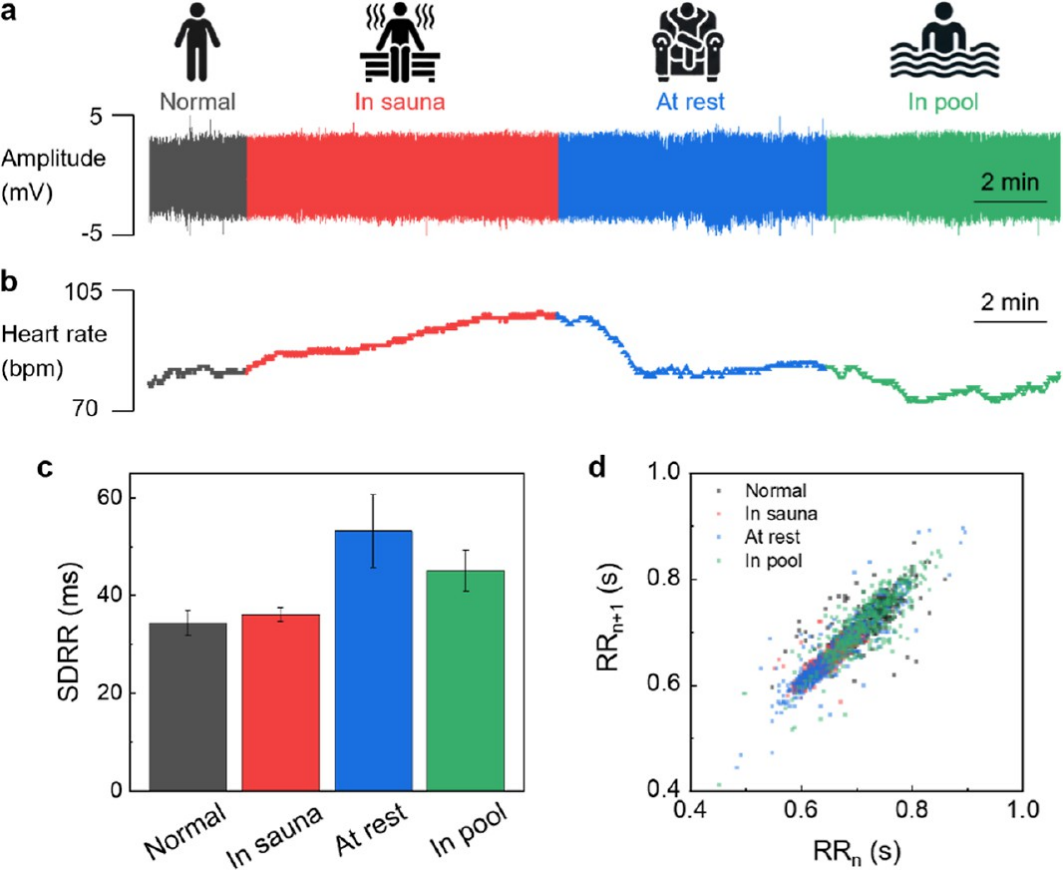
HR and HRV analysis of skin-conformal nano-electrodes. (a) Continuous ECG monitoring in different environments (normal, in a sauna, at rest, and in a pool). (b) HR monitoring based on R-peak detection. HRV analysis from (c) standard deviation of RR intervals and (d) Poincaré plot in different environments (normal, in a sauna, at rest, and in a pool).

The recorded ECG data were subsequently processed to determine HR and HRV, using the Pan-Tompkins algorithm,^41^ a widely recognized method for detecting QRS complexes in ECG signals (Figure S22). This algorithm effectively highlights the R-peaks, the most prominent features of the ECG waveform, while reducing interference from other signal components. Initially, a digital band-pass filter (5–15 Hz) is applied to the raw signals to eliminate not only the baseline wandering but also the high-frequency noise. The derivative filter emphasizes the rapid voltage changes of the QRS complex in the ECG signals. It works as a high-pass filter, amplifying the steep slopes of the QRS waves while attenuating the lower-frequency P and T waves.

Furthermore, the filter enhances the distinction between the QRS complex and other undesirable signals. The following square and convolution steps integrate the output from the derivative filter. This step smooths out false peaks that may occur within a single QRS complex and creates a waveform that more accurately represents the QRS complex’s shape and duration. With fiducial markers in place—ensuring precise timing, minimizing detection errors, and providing consistent reference points—we can reliably detect continuous R-peaks.

Based on the R-peak detection using the Pan–Tompkins algorithm, we calculated the dynamic response of HR encompassing normal conditions in a sauna, at rest, and in a pool in Figure 7b. In a normal state, the HR remained steady at around 80 bpm. Upon entering the sauna, the HR gradually increases, reaching approximately 100 bpm. After leaving the sauna and entering a resting state, the HR rapidly decreases at first, then stabilizes at around 80 bpm. Subsequently, in the pool, the HR further decreases to about 73 bpm.

To elucidate the HRV analysis, which is a measure of the irregularity or variation between consecutive heartbeats, Figure 7c exhibits the calculated standard deviation of RR intervals (SDRR) values for various conditions. RR interval is the time duration between two consecutive R waves in ECG signals and the SDRR is a measure of those variations in time RR intervals. Specifically, the SDRR values of normal, in a sauna, at rest, and in a pool exhibited 34.4, 36.1, 53.2, and 45.1 ms, respectively. These SDRR values across all circumstances fall within the normal range, which is typically 41.34 ± 14.53 ms.^42^

Finally, Figure 7d presents a Poincaré plot, a graphical tool commonly used in the analysis of HRV. The Poincaré plot is a scatter plot of RR intervals, where each interval (RR_*n*_, *x*-axis) is plotted against the following interval (RR_*n*+1_, *y*-axis). The plot’s shape and dispersion can offer both a visual and quantitative assessment of HRV by plotting consecutive RR intervals in four different conditions (normal, in a sauna, at rest, in a pool). The overall shape of the Poincaré plot was elliptical, which is a general characteristic of a healthy heart.^43^ This elongated elliptical shape indicates regular variability in RR intervals, reflecting normal HR dynamics. Therefore, the analysis of HR and HRV in different environments demonstrates the robustness of the skin-conformal nano-electrodes in practical, real-world conditions.

Moreover, the accurate and stable acquisition of R-peaks across diverse environmental conditions not only supports basic HR tracking but also enables a wide range of potentially clinically meaningful analyses. In particular, HRV derived from RR intervals has been widely adopted to assess autonomic nervous system function, mental stress levels, cardiovascular risk, and even sleep quality.^44^ These results highlight the potential of ultrathin skin-conformal nano-electrodes as a reliable platform for continuous health monitoring in demanding applications.

### 2.9 Electrophysiological Signal Monitoring in Real-Life Testing Environments

The tibialis anterior is one of the key muscles in the lower leg that plays a crucial role in understanding the muscle’s activation and coordination during the human gait cycle.^45^ For the continuous tibialis anterior EMG monitoring, we attached our skin-conformal nano-electrodes to the tibialis anterior with a 5 mm spacing between electrodes as shown in Figure 8a. As shown in Figure 8b, we tested 30 year-old human gaits at constant speeds on the treadmill. To monitor the ECG signals simultaneously, we attached skin-conformal nano-electrodes to the same position.

**Figure 8:**
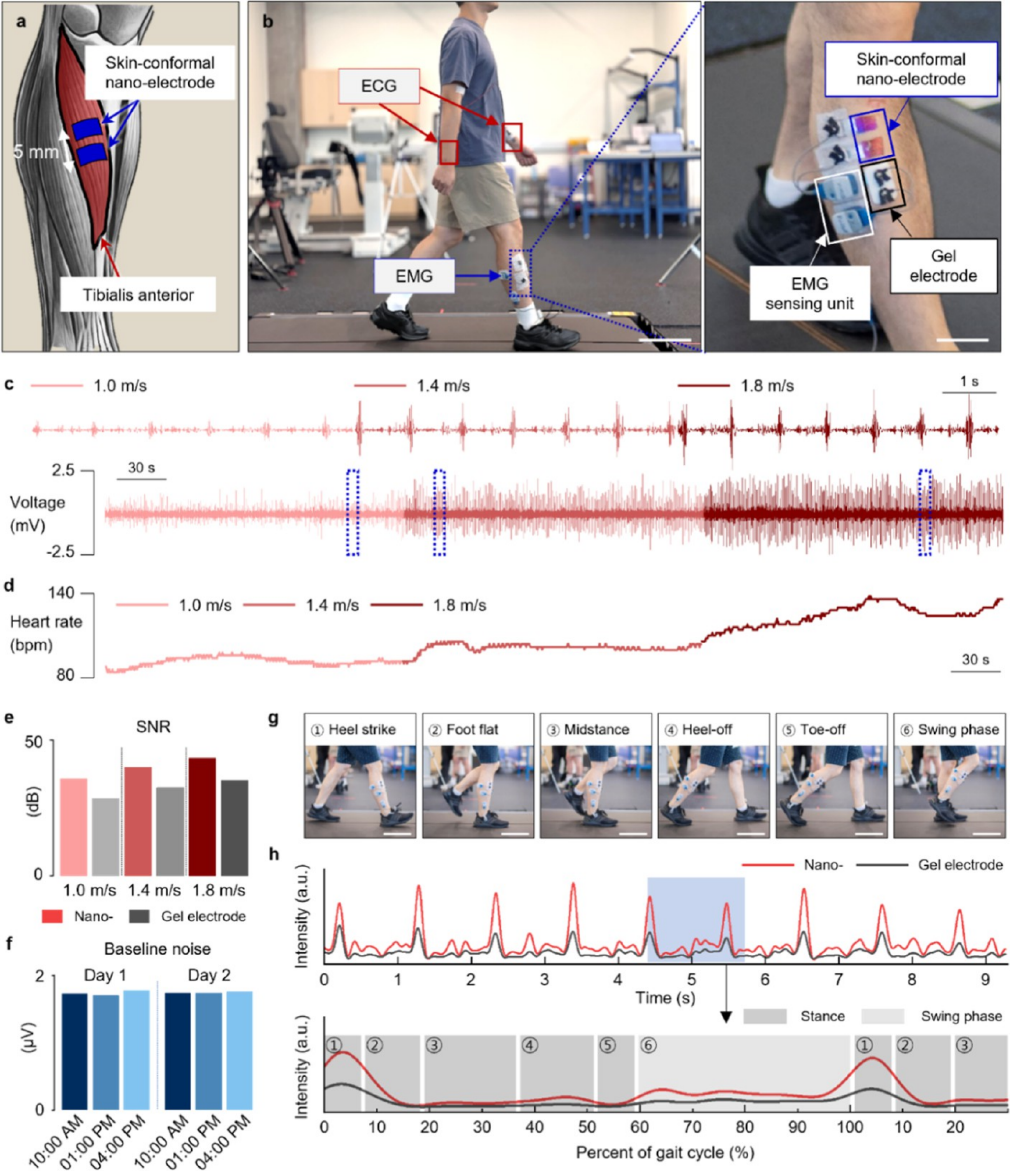
Simultaneous EMG and ECG signal monitoring during walking. (a) The skin-conformal nano-electrode attached to the tibialis anterior muscle at a distance of 5 mm. (b) Photographs of walking on the treadmill for monitoring of ECG (red) and EMG (blue) signals simultaneously (scale bar, 20 cm) and enlarged photograph of these nano- (blue), and gel (black) electrodes and sensing units (white) (scale bar, 5 cm). (c) Continuous EMG monitoring of the tibialis anterior muscle using nano-electrodes during walking at different speeds (1.0, 1.4, and 1.8 m/s). (d) Corresponding HR monitoring. (e) The SNR analysis of nano- (red), and gel (black) electrodes at three different speeds (1.0, 1.4, and 1.8 m/s). (f) The baseline noise analysis of nano-electrode over 2 days. (g) Photographs of gait cycle events matched with the corresponding natural tibialis anterior EMG patterns (scale bar, 20 cm) and (h) overlaid intensity of the envelope profile (nano- (red) and gel (black) electrodes) by analyzing two phases: stance (heel strike, foot flat, midstance, heel-off, and toe-off) and swing phases (bottom).

In addition, we also attached gel electrodes to the tibialis anterior right below the nano-electrodes, and wireless EMG sensing units were positioned next to the electrodes for simultaneous EMG signal monitoring (Figure 8b, right). For stable wireless signal transmission and continuous EMG monitoring of the tibialis anterior muscle, a mechanical gradient design ensures secure and effortless attachment to the lower leg (Figure S23). We increased the treadmill speed to 3 min intervals: 1.0 m/s (slow), 1.4 m/s (typical), and 1.8 m/s (brisk), which covers most of the walking cycles (Figure 8c, see the detailed protocol in Supporting Information Note 4).^46^ We observed that as the treadmill speed increased, the magnitude of the EMG signals increased, indicating more pronounced and periodic muscle activity during gait. Peak-to-peak voltages at low, medium, and high speeds were 1.14, 2.72, and 3.25 mV, respectively, with corresponding walking intervals of 1.08, 0.97, and 0.88 s. Additionally, HR was monitored using ECG signals, as shown in Figure 8d. HR readings increased with speed: 80 bpm at low, 100–110 bpm at medium, and 120–140 bpm at high speed, showing a strong correlation between speed and HR. These results can demonstrate how muscle and cardiovascular responses correlate with activity intensity.

Meanwhile, we compared the tibialis anterior EMG signals of our skin-conformal nano-electrodes to those of gel electrodes at three different speeds (Figure S24). In detail, at a speed of 1.0 m/s, the nano-electrodes consistently showed higher EMG signals compared to the gel electrodes (peak-to-peak voltages: 0.81 mV). The calculated SNR of the nano-electrodes were 35.6–43.4 dB, which is approximately 1.25 times higher than that of the gel electrodes across all speeds (Figure 8e). Next, we conducted long-term baseline noise analysis of nano-electrodes over 30 h as shown in Figure 8f. The initial baseline noise was approximately 1.72 μV, and only a 1% increase in baseline noise was observed even after 1 day and 6 h, highlighting the noise stability and consistent signal quality across prolonged use without causing any visible skin irritation (Figure S25).

For the in-depth analysis of muscle activity of the tibialis anterior based on the raw EMG and noise signals, the linear envelope of tibialis anterior EMG signals is essential.^47,48^ This technique helps in capturing the dynamic nature of human gaits, offering a clearer understanding of muscle function and its activation patterns during walking cycles during three successive operations (see details in Figure S26). The raw signal is cleaned from irrelevant frequencies with a high-pass filter (20 Hz) and rectified with an absolute value.

Finally, high frequencies are filtered out with a low-pass filter (6 Hz). The fundamental components of a typical gait cycle are divided into two primary phases: the stance and swing phases as shown in Figure 8g. The stance phase, which constitutes about 60% of the gait cycle, involves the foot in contact with the ground and is further divided into the heel strike, foot flat, midstance, heel-off, and toe-off phases (phases 1–5, respectively). The swing phase (phase 6), accounting for the remaining 40%, involves the foot moving through the air and is split into the leg lift and swing forward phases. These two phases constitute the fundamental mechanics of human walking. As shown in the overlaid envelope profile (Figure 8h), the skin-conformal nano-electrodes demonstrated higher signal intensity compared to the gel electrodes. Specifically, during the stance phase—particularly at the heel strike—the tibialis anterior exhibited a significant increase in EMG intensity as the muscle engaged to control dorsiflexion and prevent foot slapping.

The EMG signal intensity typically peaks during this motion phase. As the gait progressed into the foot flat-to-toe-off phases, the activity of the tibialis anterior was highly diminished, and the EMG signals dropped significantly, indicating reduced muscle engagement as the body shifted weight onto the other foot. On the other hand, during the swing phase, the tibialis anterior showed increased activity again with a lower intensity peak in the EMG signal as it lifts the foot, maintaining dorsiflexion and ensuring the toes clear the ground. Understanding the distinct stages within each phase is crucial for analyzing the muscle activity and evaluating the overall gait performance. Therefore, continuous monitoring of daily activity provides the early detection of mobility impairments, advancing the development of wearable devices for real-time health-care monitoring, diagnostics, and personalized physical therapy.

For overall analysis, we conducted a comprehensive comparison of skin-conformal nano-electrode performance with other reported electrodes, considering key factors of not only conductor and substrate types and thicknesses, encapsulation layers, and conformal contact analysis (Table S1). It included also electrophysiological performance motion artifact monitoring, underwater conditions, high temperatures, and long-term monitoring in challenging environments.^5,6,11,14,16,20,22,49–56^ A separate comparison has also been conducted for closely related skin electrodes and showed substantial improvements and is discussed further in a review (Table S2).^57^

In contrast to previously reported ultrathin electrode systems that rely on composite structures and multilayer adhesion strategies, our nano-electrode platform offers a simplified, submicron-thin architecture that maintains robust performance without additional adhesive or encapsulation layers. The unique asymmetrical surface chemistry, featuring hydrophilic MXene and hydrophobic films, facilitates skin-conformal contact durability, enabling stable signal acquisition during signal monitoring in a dynamic and underwater environment. This comparison highlights the much-enhanced performance of ultrathin nano-electrodes, particularly when contrasted with conventional micrometer-thick and electrode electrodes and under challenging conditions.

## 3 Conclusion

In summary, ultrathin amphiphilic nano-electrodes offer significantly enhanced physical conformability and resilient electrical properties, making them a robust platform for low-motion artifact and water-resistant electrophysiological monitoring. The unique design of dual wettability, incorporating a hydrophilic MXene conductor for enhanced skin adhesion and a hydrophobic cross-linked supporting polymer layer with hydrophobic properties, ensured strong contact with the skin, even during intense motions in air and underwater. Our analytical model and structural analysis demonstrated that a nano-electrode provides strong highly adherent physical conformal contact with the skin, effectively adapting to the intricate complex textures of human skin.

These ultrathin skin-conformal nano-electrodes demonstrated outstanding mechanical durability and electrical stability, maintaining conformal contact and negligible resistance variation even after extensive bending/stretching cycles and days of attachment. Furthermore, the nano-electrodes exhibited low and stable skin interfacial impedance in air and underwater, ensuring reliable and high-fidelity signal collection. Hence, they exhibited a high to noise ratio, lower baseline noise, and, most importantly, extremely high suspension of motion artifacts under challenging environments, including those in a sauna and pool. We further demonstrated the effective use of skin-conformal nano-electrodes for concurrent EMG and ECG monitoring during treadmill walking with excellent long-term stability, especially for detecting complex muscle activities.

The comprehensive characteristics of conformability, water resistance, and long-term stability enable reliable, high-fidelity electrophysiological skin signal acquisition, far surpassing traditional thicker electrodes across various conditions and environments. These outcomes were supported by an extensive series of structural and durability validations as well as repeatable mechanical deformation, water immersion, and sweat exposure tests. Collectively, these evaluations affirm the nano-electrodes’ robust mechanical and environmental resilience, making them uniquely well-suited for sustainable, long-term bioelectronic applications for continuously monitoring mobility in daily active life, offering promising wearable applications for real-time healthcare, diagnostics, and performance monitoring.

## 4 Experimental Section

### 4.1 Preparation of MXene (Ti_3_C_2_T_*x*_) Nanosheets

The Ti_3_AlC_2_ MAX phase was synthesized using high-purity TiC, Al, and Ti powders (Alfa Aesar, USA) combined in a 2:1:1 atomic ratio of TiC/Ti/Al.^58^ The mixture underwent ball milling for 18 h using zirconia balls at 60 rpm, with a 2:1 ball-to-powder ratio. The milled powder was then heated in a tube furnace under flowing argon (200 cm^3^/min) at 1400 °C for 2 h, with 3 °C/min heating and cooling rates. The resulting material was milled with a TiN-coated bit and sieved to obtain particles smaller than 38 μm. Then a 1 L reactor was employed for the etching process. A mixture of HF, water, and HCl (50:150:300 mL) was prepared, followed by gradual addition of 50 g of Ti_3_AlC_2_ powder over 5 min using a screw feeder. The mixture was stirred for 24 h at 150 rpm while maintaining 35 °C through water cooling. After etching, the product was repeatedly washed via centrifugation at 3500 rpm until achieving neutral pH. The MXene powder was then delaminated by mixing with 1 L of deionized water and 50 g of LiCl, stirring for 24 h at 150 rpm. Multiple washing cycles were performed until achieving a stable colloidal solution. The final solution underwent extended centrifugation to remove any remaining multilayer particles, and the stable MXene suspension was vacuum filtered through Celgard membranes (64 nm pore size, 3501 coated polypropylene) to produce free-standing films.

### 4.2 Fabrication of Skin-Conformal Nano-Electrodes

Glass substrates were cleaned with DI water, acetone, and isopropyl alcohol under sonication for 10 min each and coated with a Micro-90 (Cole-Parmer, USA) sacrificial layer. Then, parylene films were deposited by chemical vapor deposition using a parylene coater (SCS Labcoater PDS 2010, Specialty Coating Systems, USA). Subsequently, the parylene films were treated with O_2_ plasma to prepare hydrophilic surfaces. A PLL solution was spin-coated (0.1 w/v % in H_2_O, Sigma-Aldrich, USA) at 2000 rpm for 60 s, and then an MXene solution (25 mg mL^–1^) was added at 2000 rpm for 60 s, following which the system was annealed at 110 °C to remove DI water for 10 min. The as-fabricated skin-conformal nano-electrode was then dissolved with the parylene substrate on micro-90 and glass in DI water to remove the micro-90 sacrificial layer. The total thickness of the nano-electrode and PLL layer is approximately 350 and 3 nm, respectively (Figure S27).

### 4.3 Transfer Process of Skin-Conformal Nano-Electrodes onto Human Skin

The as-fabricated skin-conformal nano-electrode on a glass substrate was placed in water. The electrode detached from the glass substrate as the Micro-90 sacrificial layer was dissolved into the water. The hydrophobicity of the parylene layer and the hydrophilicity of the MXene layer caused it to float on the surface of the water, with the parylene layer facing the air and the MXene layer in contact with the water. Then, the electrode was mounted onto human skin or commercial artificial replica skin (silicone practice fingers, DECINIEE BEAUTY CO.) by dipping the body parts, where the signal will be measured, into water and positioning it under the film. By lifting it, the electrode was mounted on the skin, removing residual water, and leaving it dry for 10 min before electrophysiological measurement.

### 4.4 Electrical Characterization

The skin–electrode interfacial impedance was measured by an LCR meter system (4284A, Agilent Technologies, USA) with a range of 20 Hz to 100 kHz. The measurements were carried out by attaching 2 × 2 cm size electrode pairs with a center-to-center distance of 5 cm on the forearm. The resistance changes during forward and backward bending motions were measured with a semiconductor analyzer (E5272A, Agilent Technologies, USA). The nano- and thick electrodes cut by 3 cm × 2 cm were attached to the inner wrist, and Nickel/Copper fabric conductive tapes were connected to both ends of the electrodes.

### 4.5 Evaluation of ECG and EMG Signals of Skin-Conformal Nano-Electrodes

To investigate the real-time monitoring of ECG and EMG signals, nano- and thick electrodes were fabricated by a rectangular shape (3 cm × 2 cm) and compared with commercial Ag/AgCl gel electrodes (Red Dot, 3M Company) for their performance in the comparison of robustness against motion artifacts, stability underwater, and durability over time some. The electrodes were connected with a commercial wireless electrophysiological data acquisition device (BioRadio, Great Lakes NeuroTechnologies, USA). For ECG signal acquisition, two measurement electrodes were mounted on the wrists with the reference electrode placed on the back of the hand. The electrical sheet resistance of the MXene conductors on the nano- and thick electrode was controlled at 50 Ω·sq^–1^ and the ECG signals were measured in an accessible-SNR-frequency domain up to 2 kHz. The SNR of electrophysiological electrodes was calculated as follows: (see details in Supporting Information Note 3):

4 $$SNR=20\log\left(\frac{V_{rms,signal}}{V_{rms,noise}}\right)$$

where *V*_rms,signal_ is the RMS value of the signal (frequency range: 0.5–100 Hz) and *V*_rms,noise_ is the RMS value of the noise (frequency range: >100 Hz). For EMG signal acquisition, two measurement electrodes were mounted on the forearm to measure the brachioradialis muscle signals during the act of grabbing various objects and motions. The collected data were wirelessly transmitted to a computer for further data analysis. The sampling rate for all signals was 2 kHz. Data analysis was performed with Matlab software (Mathworks, USA). For EMG signal acquisition while walking on the treadmill, another wireless electrophysiological data acquisition device (Ultium EMG, Noraxon, USA) was used to attach the acquisition system on the calf securely. The skin-conformal nano-electrodes and commercial gel (Dual EMG electrodes, Noraxon, USA) electrodes were attached to the subject’s tibialis anterior muscle. All ECG data were filtered by a high-pass filter (0.5 Hz, Butterworth, third) and all EMG data were filtered by a high-pass filter (10 Hz, Butterworth, third). The signal qualities were quantified with RMS and SNR signals. Detailed procedures of ECG and EMG analysis are depicted in Supporting Information Note 3.

### 4.6 Characterization

Contact angles of MXene and the parylene surface were characterized with an optical contact angle meter (KSV CAM 101) and a CCD camera (DMK 23U618, Imaging Source). Optical images were obtained using a digital optical microscope (10″ HDMI LCD Digital Microscope 1500X, Dcorn, USA), characterized the morphology of the skin-conformal nano-electrodes by field emission SEM (SU-8230, Hitachi, Japan), and determined the thickness of the MXene conductor by atomic force microscopy (Dimension Icon AFM, Bruker, USA) in accordance with the usual procedure.^59^ A profilometry height profile of skin-conformal nano-electrodes on the artificial skin was characterized by an optical surface profiler (VK-X3000, Keyence, USA).

The optical transmittance of the skin-conformal nano-electrodes was measured using a UV–vis spectrophotometer (UV-3600 Plus, Shimadzu, Japan). The zeta potential of the MXene and PLL was analyzed by a Zetasizer device (Nano ZS, Malvern, UK). All measurements were repeated on at least five independently fabricated samples for each condition to ensure reproducibility.

## Supplementary Material

















## Data Availability

The data that support the findings of this study are available from the corresponding author upon reasonable request.

## Notes

Theoretical modeling of adhesion energy and bending stiffness; ECG/EMG signal analysis methods; testing protocols in varying environments; fabrication and transfer steps; SEM/AFM images; adhesion properties; mechanical durability; breathability; biocompatibility; signal quality comparisons; signal processing; and performance comparison of nano-electrodes against prior systems (PDF)
Transferprocess of nano-electrodes onto human skin (MP4)
Water-repellent properties of nano-electrodes (MP4)
Sustainability of water flow and rubbing in water (MP4)
Removal of skin-conformal nano-electrodes using a soap (MP4)
Continuous EMG monitoring in the air (MP4)
Continuous EMG monitoring in underwater (MP4)
Baseline stability under the deformations (MP4)
