# Supplementary material for: Robust Skin-Conformal Nano-Electrodes for Sustainable Health and Performance Monitoring
Source: ACS Nano. 2025 Aug 12;19(33):30322–37. doi: 10.1021/acsnano.5c08540 (PMC12392733; doi:10.1021/acsnano.5c08540)
Supplement: Supplementary file 1 [file nn5c08540_si_001.pdf]

Supplementary Information

Robust Skin-Conformal Nano-Electrodes for Sustainable Health and Performance Monitoring

*Jinyoung Kim[1]†, Sehyun Park[1]†, Jisoo Jeon[1], Dong-hee Kang[1], Gwendolyn M. Bryan[2,3], Timothy J. Broderick[2], Morley Stone[2], Vladimir V. Tsukruk[1]**

[1] School of Materials Science and Engineering, Georgia Institute of Technology, Atlanta, Georgia, 30332, United States

[2] Institute for Human and Machine Cognition, Pensacola, Florida, 32502, United States

[3] Department of Intelligent Systems and Robotics, University of West Florida, Pensacola, Florida, 32514, United States

Supplementary Video 1. Transfer process of nano-electrode onto human skin

Supplementary Video 2. Water-repellent properties of skin-conformal nano-electrodes

Supplementary Video 3. Sustainability of water flow and rubbing in water

Supplementary Video 4. Removal of skin-conformal nano-electrodes using soap

Supplementary Video 5. Continuous EMG monitoring of nano-electrodes in the air

Supplementary Video 6. Continuous EMG monitoring of nano-electrodes in underwater

Supplementary Video 7. Baseline stability under the deformations (pinch, torsion, spread, and touch)

Supplementary Note 1. Theoretical analysis of the work of adhesion for the critical thickness of parylene layer toward conformal contact.

To secure the electrode's conformability on the skin and avoid mechanical failure, the maximum thickness (critical thickness) that allows conformal contact on human skin must be considered and evaluated here.

1) Bending-induced strain energy per area

Our developed electrode system consists of two layered materials: MXene and Parylene. In our analysis, we first derive the bending-induced strain energy per area, which is plotted as a red line in **Figure 1e**. Second, we derive the work of adhesion, represented by a dotted line in the same figure. For clarity in our calculations, we use subscript annotations: 1 for MXene and 2 for parylene.

First, for the bending-induced strain energy per area derivation, we fixed the MXene layer thickness of 50 nm (t_1), thus we analyzed the critical thickness of parylene (t_2) where the conformal contact occurs. To deform along with the curvilinear skin surface, the work of adhesion (γ) of the electrode needs to be higher than the strain energy of the electrode.[1]

The bending-induced strain energy per area of the electrode is calculated as:[2,3]

$$\frac{U_{bending-strain}}{A} = \frac{E_{eff}t^3}{24R^2} \quad (1)$$

$$E_{eff} = \sum_{i=1}^{N} \frac{E_i t_i}{t_{total}} \quad (2)$$

where $U_{bending\text{-}strain}$ is bending-induced strain energy, A is an area of the device, E_{eff} is the effective elastic modulus of the electrode, and E_{eff} is calculated as a composite of N layers, t is the total thickness of the electrode, R is the radius of curvature, E_i is the young's modulus of ith layer, t_i is the thickness of ith layer. R was considered to be approximately 1 mm for skin[4].

2) Work of adhesion

To obtain the work of adhesion on soft human skin as a function of the parylene thickness, the skin surface was assumed as a sinusoidal model, which is described as:

$$y(x) = \frac{h_{rough}}{2}\left(1 + \cos\frac{2\pi x}{\lambda_{rough}}\right) \quad (3)$$

where y(x) is skin roughness, h_{rough} is roughness amplitude, and λ_{rough} is wavelength.[5]

Conformal contact induces exact match between the electrode and skin surface. Therefore, electrode displacement ($w(x)$) and skin displacement ($u_z(x)$) are given as:

$$w(x) = \frac{h}{2}\left(1 + \cos\frac{2\pi x}{\lambda_{rough}}\right) \text{ (4)}$$

$$u_z(x) = y - w = \frac{h_{rough} - h}{2}(1 + \cos\frac{2\pi x}{\lambda_{rough}}) \text{ (5)}$$

The maximum deflection of the electrode is h:

$$h = \frac{E_{skin} h_{rough}}{\frac{16\pi^3 EI}{\lambda_{rough}^3} + E_{skin}} \text{ (6)}$$

Conformal contact conditions are defined by calculating interfacial contact energy ($U_{conformal}$) by:

$$U_{conformal} = U_{bending} + U_{skin} + U_{adhesion} \text{ (7)}$$

Electrode bending energy, skin elastic energy, and contact adhesion energy are denoted as $U_{bending}$, U_{skin}, and $U_{adhesion}$, respectively.

Bending energy is calculated as:

$$U_{bending} = \frac{1}{\lambda_{rough}} \int_0^{\lambda_{rough}} \frac{EI(w'')^2}{2} dx = \frac{\pi^4 EIh^2}{\lambda_{rough}^4} \text{ (8)}$$

The MXene and parylene are a layered structure where the effective bending stiffness (EI) is represented as:

$$EI_{total} = EI_{MXene} + EI_{parylene} \text{ (9)}$$

$$EI = \sum_{i=1}^{N} E_i t_i \left[\left(b - \sum_{j=1}^{i} t_j\right)^2 + \left(b - \sum_{j=1}^{i} t_j\right) t_i + \frac{1}{3} t_i^2\right] \text{ (10)}$$

where

$$b = \frac{\sum_{i=1}^{N} E_i t_i \left(\sum_{j=1}^{i} t_j - \frac{1}{2} t_i\right)}{\sum_{i=1}^{N} E_i t_i} \text{ (11)}$$

E_i is the elastic modulus of the ith layer.

The elastic energy of skin is represented as:

$$U_{skin} = \frac{1}{\lambda_{rough}} \int_0^{\lambda_{rough}} \frac{\sigma_z u_z}{2} dx = \frac{\pi E_{skin}\left(h_{rough} - h\right)^2}{16\lambda_{rough}} \quad (12)$$

Where the normal stress of the skin surface is:

$$\sigma_Z = \frac{\pi E_{skin}\left(h_{rough} - h\right)}{2\lambda_{rough}} \cos\frac{2\pi x}{\lambda_{rough}} \quad (13)$$

Adhesion energy is calculated as:

$$U_{adhesion} = -\gamma \int_0^{\lambda_{rough}} \sqrt{1 + (w')^2}\, dx \approx -\gamma\left(1 + \frac{\pi^2 h^2}{4\lambda_{rough}^2}\right) \quad (14)$$

$U_{conformal} = 0$ when the conformal contact occurs. Substituting into energy equation and solving for γ produces:

$$\gamma = \frac{\left(\frac{\pi^4 \cdot EI \cdot h^2}{\lambda_{rough}^4} + \frac{\pi \cdot E_{skin}\left(h - h_{rough}\right)^2}{16\lambda_{rough}}\right)}{\frac{\pi^2 \cdot h^2}{4\lambda_{rough}^2} + 1} \quad (15)$$

$$h = \frac{E_{skin} \cdot h_{rough}}{\frac{16\pi^3 \cdot EI}{\lambda_{rough}^3} + E_{skin}} \quad (16)$$

In this study, skin conditions and material properties are h_{rough} = 25.7 µm, λ_{rough} = 331.7 µm, E_{skin} = 130 kPa, h_1=50 nm, A=6 mm^2 E_1= 330×10^9 E_2=3.8×10^9.[6–8]

In summary, we obtained the work of adhesion as a function of parylene thickness (t_2). The work of adhesion, as described by Eq. (15), is larger than the bending-induced strain energy per area of the electrode given by Eq. (1). These two values converge at a parylene thickness of 300 nm when the electrode structure makes conformal contact, as specified in **Figure 1e**. Below this thickness, conformal contact is theoretically achievable and for higher thickness mismatch of the topographies might happen. Therefore, it is essential to maintain the parylene thickness thinner than 300 nm to ensure conformality to epidermal topography and reduce the delamination risks as discussed in the main text.

Supplementary Note 2. Comparative analysis of theoretical bending stiffness: Nano-Electrodes versus Human Skin.

Using Equation (9) in **Supplementary Note 1,** Supporting Information, the bending stiffness of the nano-electrode is calculated to be 2.1×10^{-12} N·m. The bending stiffness of skin (EI_{skin}) can be calculated using the following equation:

$$EI_{skin} = \frac{E_{skin} \cdot t_{skin}^3}{12 \cdot (1-\nu^2)} \quad (17)$$

where E_{skin} = 130 kPa, t_{skin} = 1.5 mm, and ν = 0.48 is the Poisson's ratio of the skin.[9,10]

The resulting calculation is $EI_{skin} = 3.8\times10^{-5}$ N·m, which is approximately 1.8×10^{7} times stiffer than the nano-electrode.

Supplementary Note 3. ECG and EMG measurement modes.

1) ECG

To quantify and analyze the quality of ECG signals from various electrodes, we first obtained the power spectral density estimate using Welch's method in MATLAB ('pwelch' command). The parameters for the pwelch command were set to a 2000-point Hanning window with 50% overlap.[11] The frequency range of interest for ECG signals is typically below 100-120 Hz.[12,13] To remove baseline wandering, we filtered out frequencies below 0.5 Hz. As shown in Figure S11, the region between 0.5-100 Hz (highlighted in blue) was considered to represent the collected signal in our quality analysis. We extracted noise from the region shown in red (100-1000 Hz), chosen because it lies explicitly in the noise floor.[14] The noise level was then quantified using the root-mean-square value.

2) EMG

We used the same 'pwelch' command to analyze the quality of the EMG signal from the gripping test as the ECG signal above. To remove baseline wandering, we filtered out frequencies below 10 Hz for the EMG signal.[15] Unlike the ECG signal, a subject can control the EMG signal. The signals from the idle state (when the subject does not give any activity on muscle) were considered as noise and root-mean-squared for further analysis. Then, the following formula was used to convert the ratio of the signal and noise to power in dB:

$$SNR = 20\log\left(\frac{V_{rms,signal}}{V_{rms,noise}}\right) \quad (18)$$

where $V_{rms,signal}$ is the RMS value of the signal, and $V_{rms,noise}$ is the RMS value of the noise.

Supplementary Note 4. Detailed experiment procedures for Figure 7 and 8.

1) Heart rate monitoring in different environments (Normal-In sauna-At rest-In pool)

Real-time ECG data was collected over a 25-min period to observe heart rate changes under various conditions. The experiment consisted of four phases - Normal (2 min), In sauna (10 min), At rest (7 min), and Pool (6 min). In the first phase, after the mounted ultrathin electrodes completely dried, the participant sat on a chair at room temperature to establish a baseline heart rate in idle conditions. Heartrate in this state is referred to as 'Normal'. Next, the volunteer entered an 80 °C dry sauna room within 10 min. This phase aims to observe the increase in heart rate due to heat stress. Following the sauna, in 'At rest' condition, the volunteer returned to the same conditions as in the 'Normal' phase for 7 min. This allowed the heart rate to decrease and return to a normal state. Finally, the volunteer entered a pool with a water temperature of 30 °C for 6 min to decrease heart rate due to the calming effect of water immersion. The experiment was designed to study how the volunteer's heart rate responded to different environmental conditions, including high-temperature heat stress and a comfortable swimming environment.

2) Tibialis anterior monitoring during treadmill walk

The experiment involved attaching two sets of electrodes to the participant's right leg tibialis anterior: a pair of ultrathin electrodes and a pair of gel electrodes. After the mounted ultrathin electrodes completely dried, the participant then performed a walking test on a treadmill, maintaining speeds of 1.0, 1.4, and 1.8 m/s for 3 min each, in consecutive order to monitor the EMG signal from the pairs of the electrodes sets. This walking protocol was repeated six times over the course of two days to analyze long-term performance of both electrodes. The participants kept wearing the electrodes sets over two days to analyze their endurance during daily activities. On each day, the tests were conducted at three different times: 10 am, 1 pm, and 4 pm.

Supplementary Figures

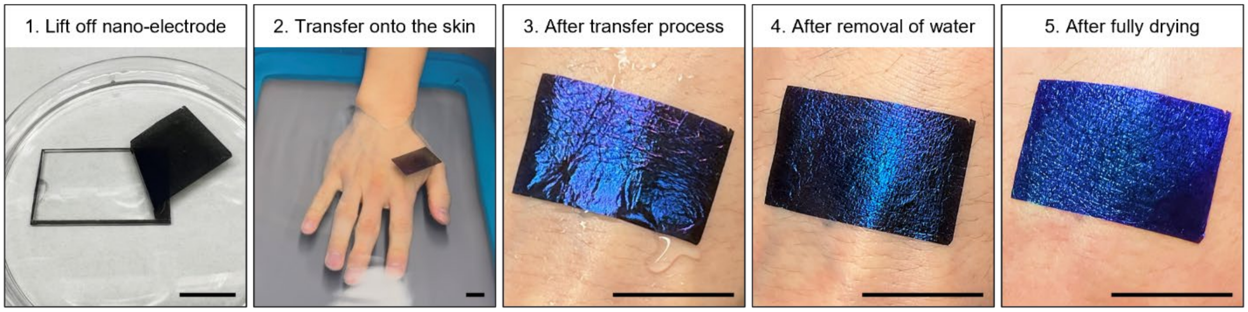

Figure S1. Photographs of the transfer process of nano-electrodes onto human skin (Scale bar, 2 cm).

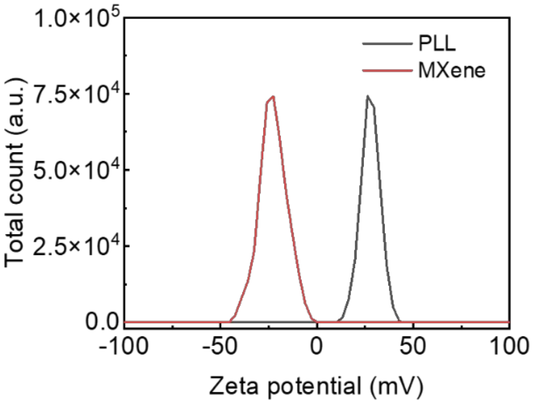

Figure S2. Zeta potential of aqueous MXene and 0.1 wt% PLL solutions.

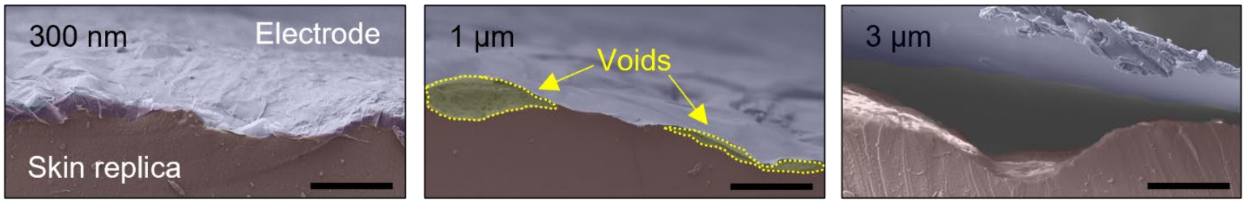

Figure S3. Cross-sectional SEM images of conformal contact of the skin-conformal nano-electrodes with 0.3-, 1-, and 3-µm-thick parylene electrodes on the skin (scale bar, 100 µm).

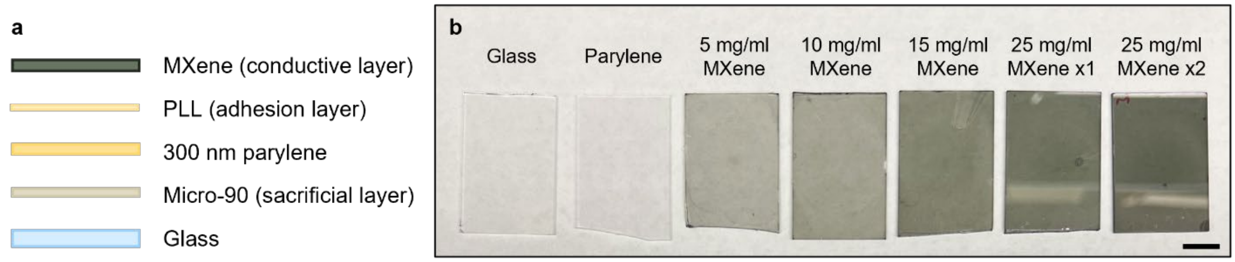

Figure S4. a) Design of the multilayered skin-conformal nano-electrodes b) Photographs of the skin-conformal nano-electrodes with different concentrated MXene solutions (5-25 mg/mL) (scale bar, 1 cm).

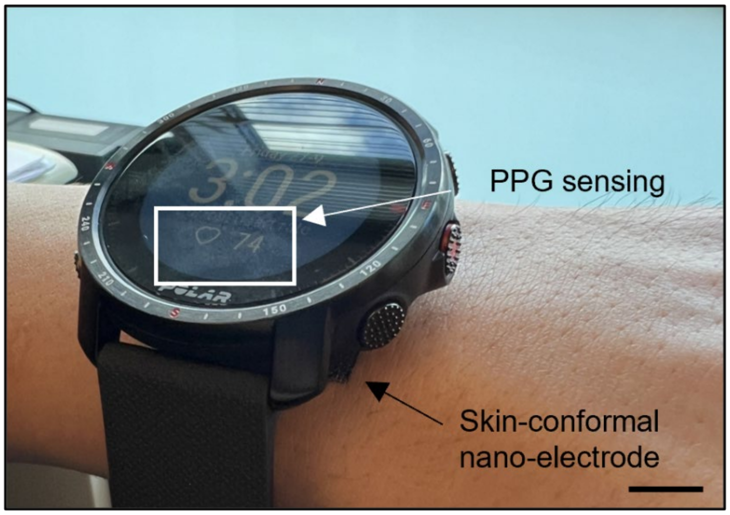

Figure S5. Combining a commercial PPG sensor on the skin-conformal nano-electrodes with BPM monitoring not affected by the presence of the sensor beneath the watch (Scale bar, 1 cm).

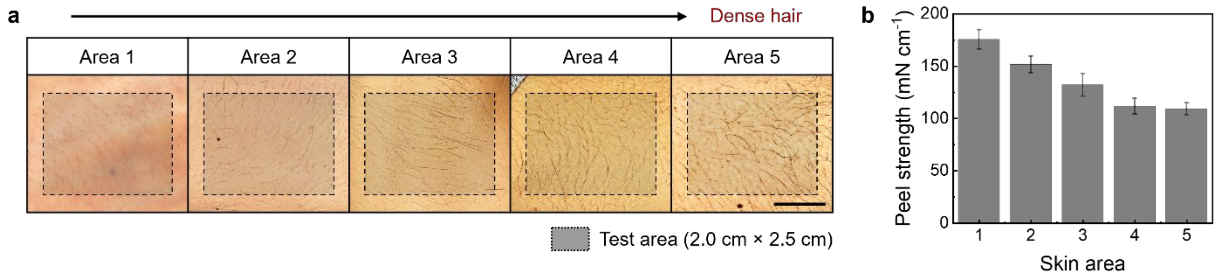

Figure S6. (a) Photographs of skin-conformal nano-electrodes applied to human skin with varying hair densities (scale bar: 10 mm). (b) Peel strength test of nano-electrodes with different thicknesses delaminated from the artificial skin (peeling rate of 50 mm/min).

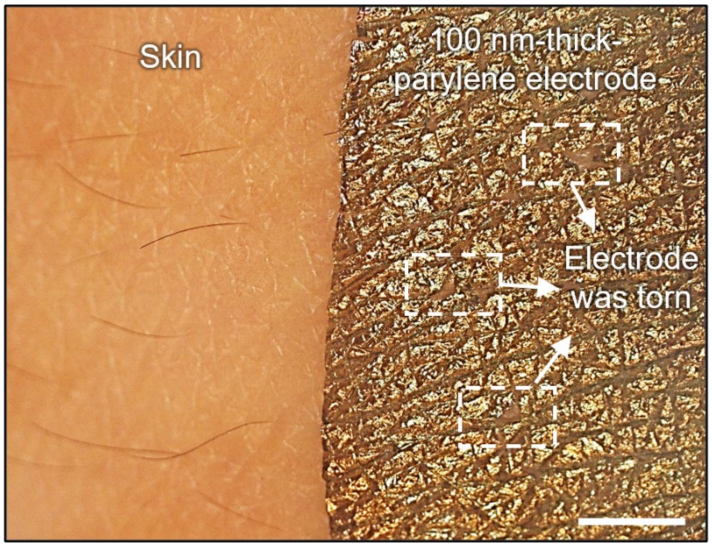

Figure S7. Optical image of the conformal contact using 100 nm-thick-parylene electrodes on the skin (scale bar, 1 mm).

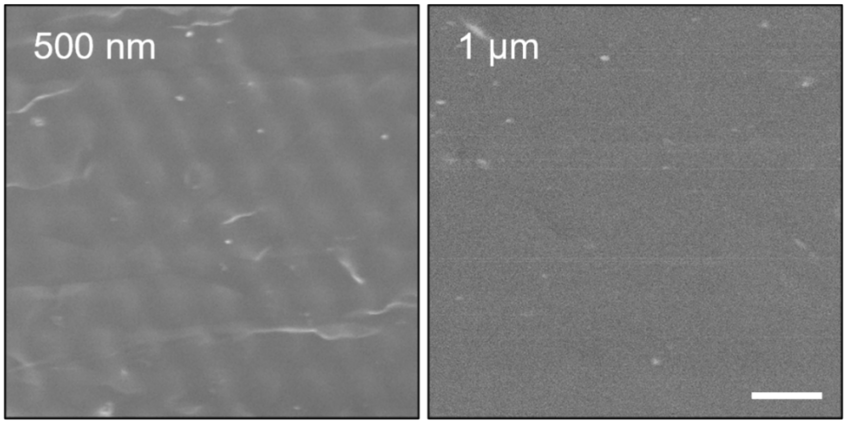

Figure S8. SEM images of the 0.5 and 1-µm-thick parylene electrodes coating the dome-like patterned array (scale bar, 10 µm).

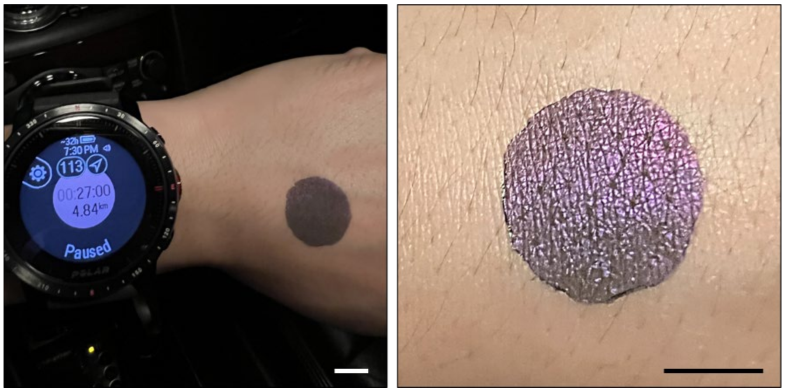

Figure S9. Photographs of the skin-conformal nano-electrodes on the skin after 5 km running (Scale bar, 1 cm).

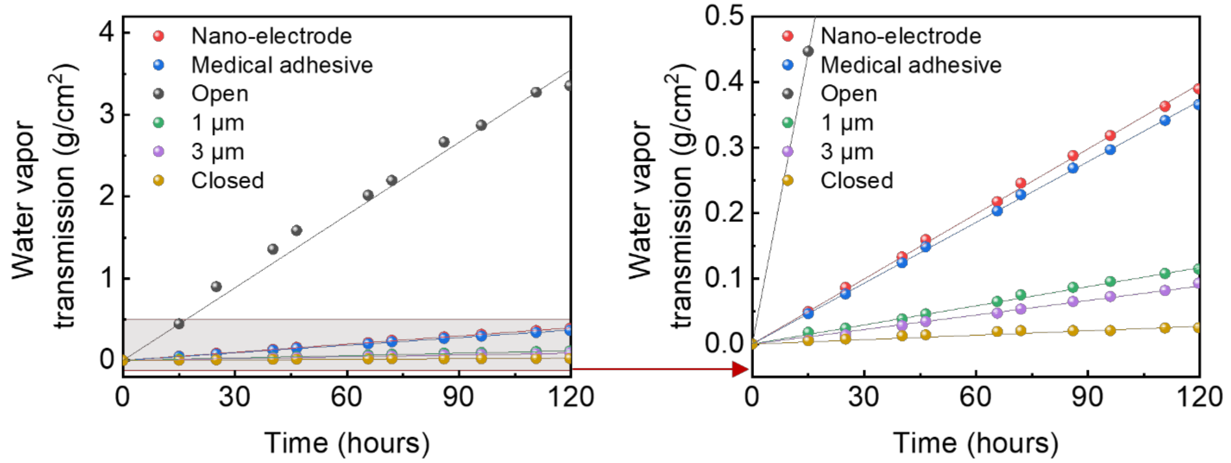

Figure S10. Water vapor transmission through the electrodes different parylene thicknesses (300 nm- (*red*), 1 μm- (*green*), and 3 μm- (*purple*) parylenes), and commercial medical adhesive film (*blue*) under relative humidity 20 ± 10% at 35 °C.

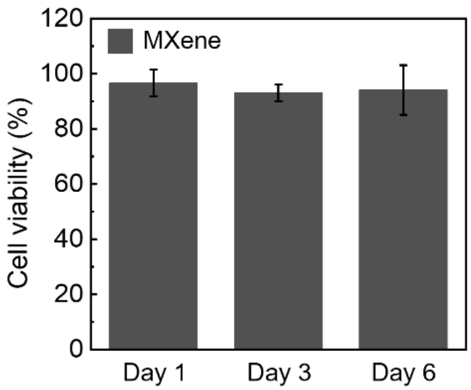

Figure S11. Evaluation of cell compatibility for the biocompatibility of skin-conformal nano-electrodes.

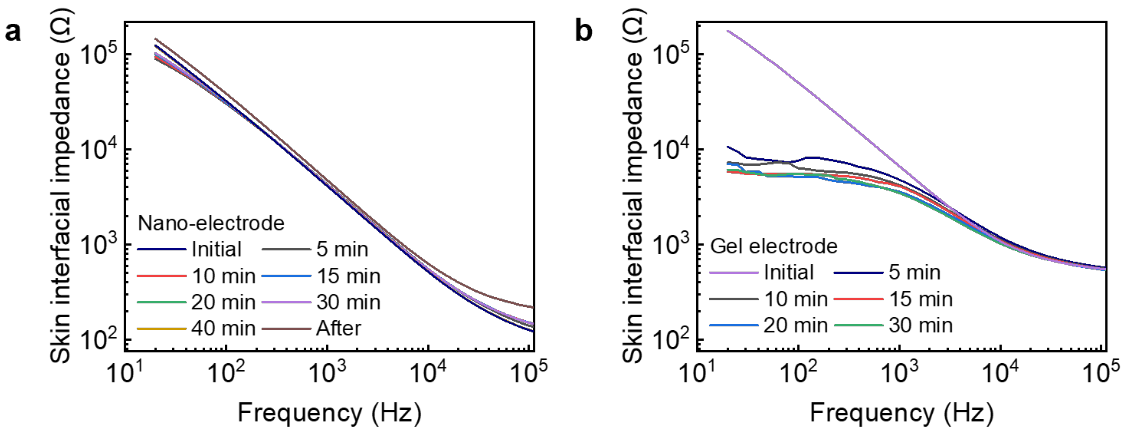

Figure S12. Skin interfacial underwater impedance at different times for a) nano- and b) gel electrodes.

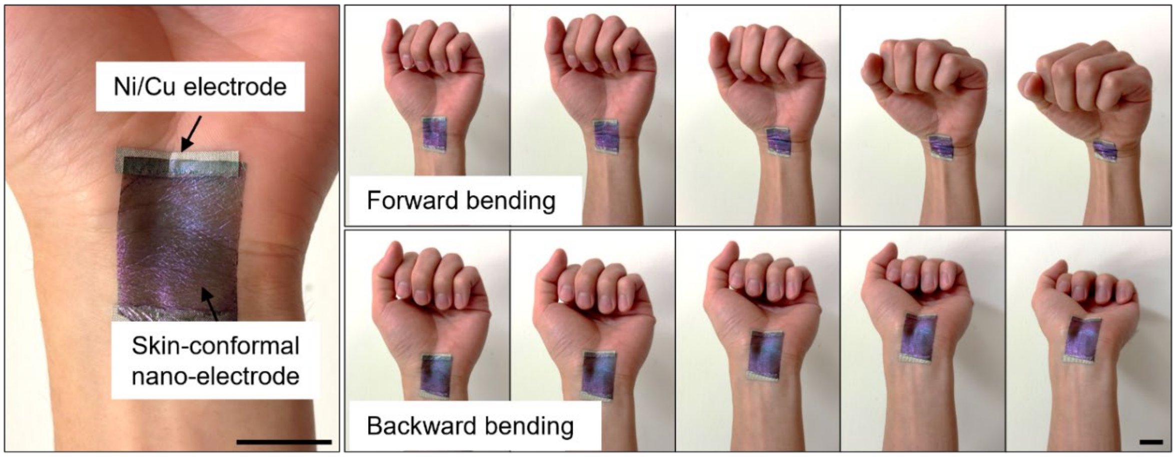

Figure S13. Photographs of skin-conformal nano-electrodes attached to the inner wrist subjected to forward and backward bending (scale bar, 2 cm).

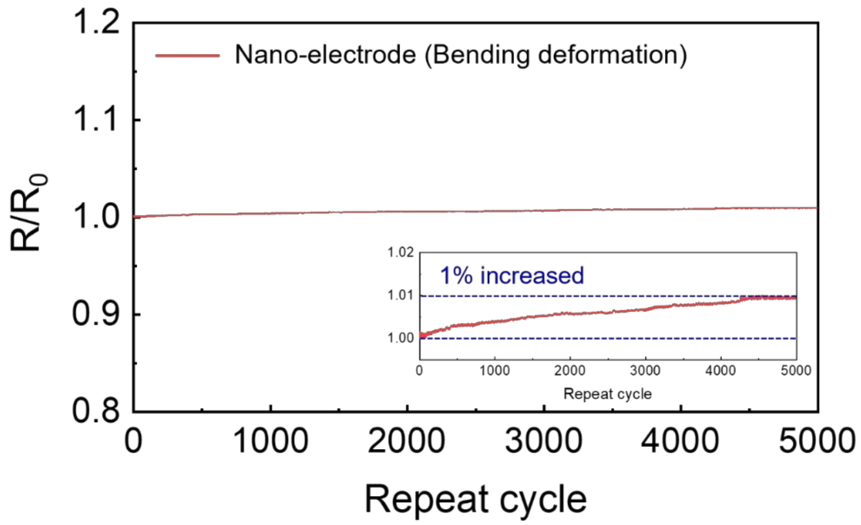

Figure S14. Effects of repeated bending deformations on the resistance change of skin-conformable nano-electrodes (5,000 cycles, curvature radius: 5 cm).

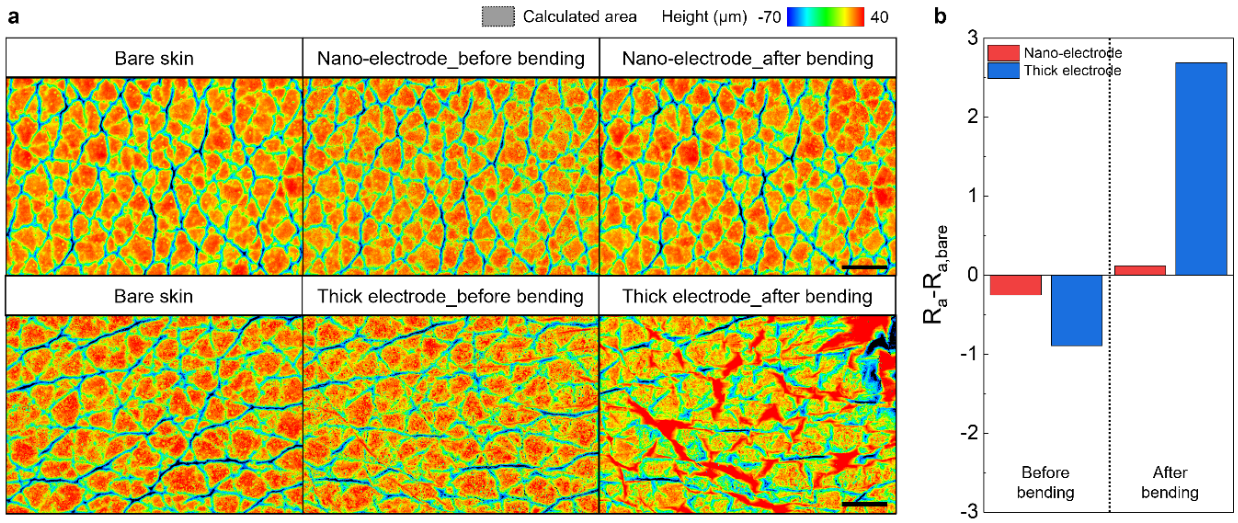

Figure S15. Effects of compressive bending deformations (radius of curvature, 5 cm, 1,000 bending cycles) on change in profilometry height profiles for nano- and thick parylene electrodes. (a) Height profile images for nano- (*top*) and thick (*bottom*) parylene electrodes bare skin (*left*), before (*middle*), and after (*right*) compressive bending (scale bar, 2 mm). (b) The roughness analysis of 3D areal surface (area: 9 mm × 12 mm).

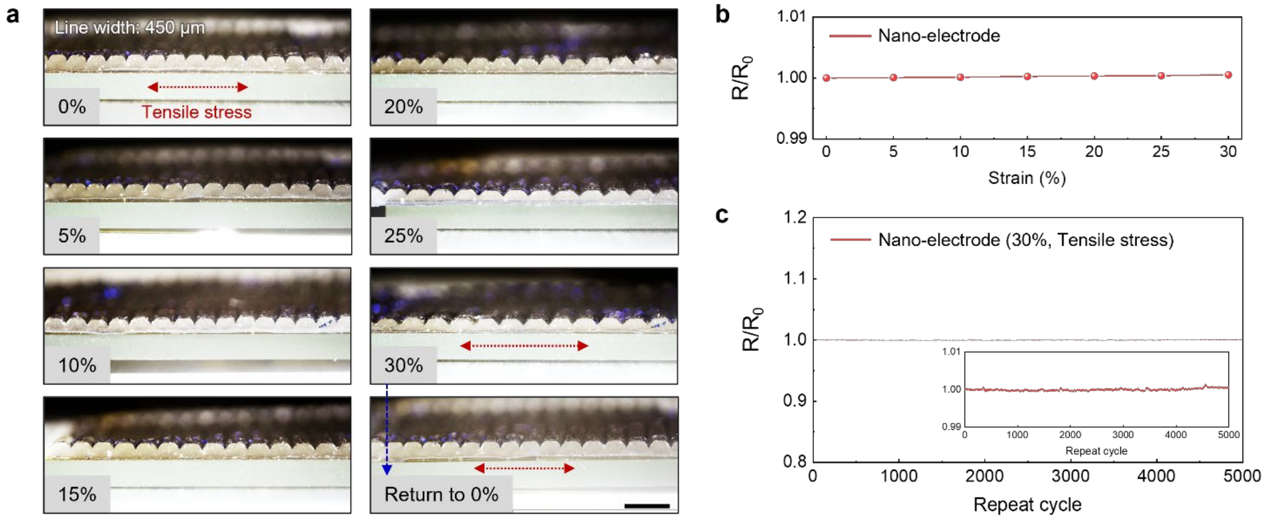

Figure S16. Effects of tensile stress (30% strain) on change in resistance to skin-conformal nano-electrodes (scale bar, 1 mm). (a) Optical images of stretched nano-electrode on silicone elastomer substrate (Ecoflex 00-30). (b) Relative resistance variations of nano-electrode up to 30% strain. (c) Repeated tensile stress on the resistance change of the nano-electrodes over 5,000 cycles.

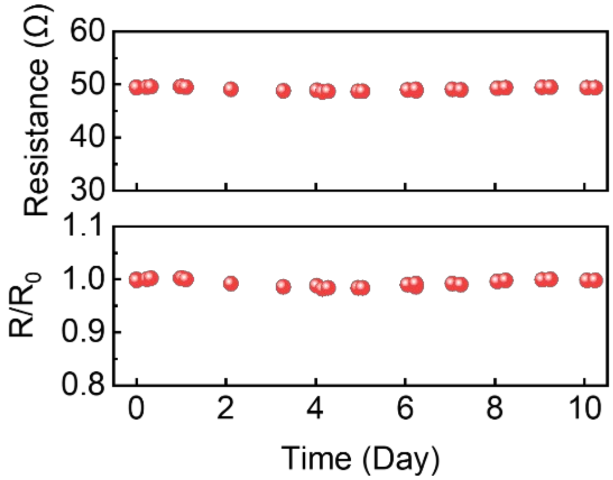

Figure S17. Long-term (10-days) electrical stability of the skin-conformal nano-electrodes. Nano-electrodes (size: 3 cm × 2 cm) were affixed to the artificial skin, with conductive ink on medical tape used to connect to both ends of the electrodes.

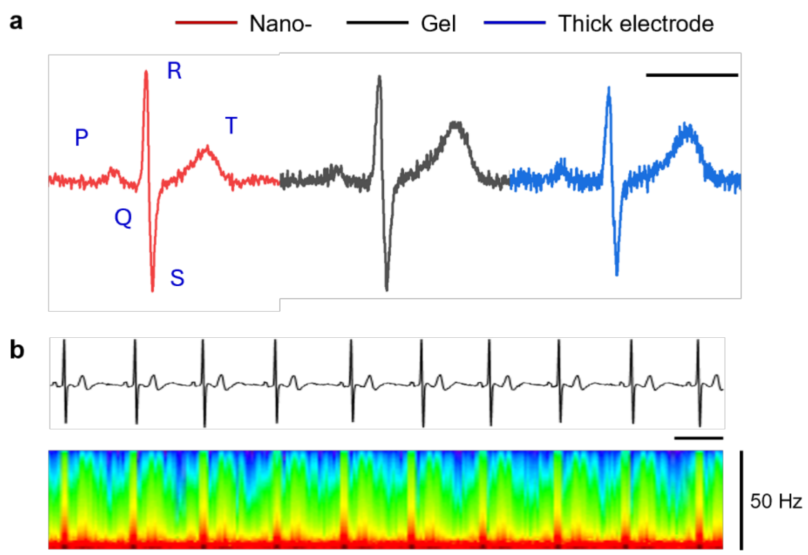

Figure S18. a) Comparison of PQRST waveforms of the SNR analysis of nano- (*red*), gel (*black*), and thick (*blue*) electrodes (scale bar, 0.5 s). b) Short-time Fourier transform spectrogram up to 50 Hz of the nano-electrode (scale bar, 1 s).

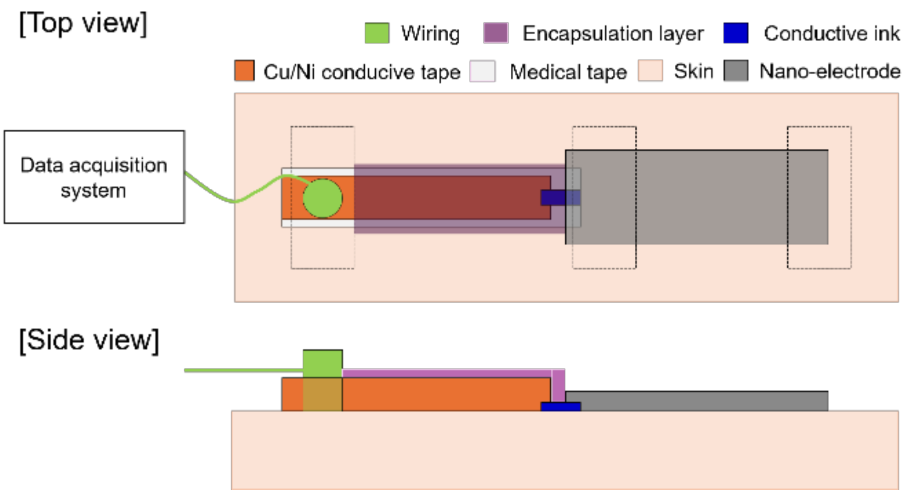

Figure S19. The schematic of interface design between the nano-electrode and the interconnected electrical circuit.

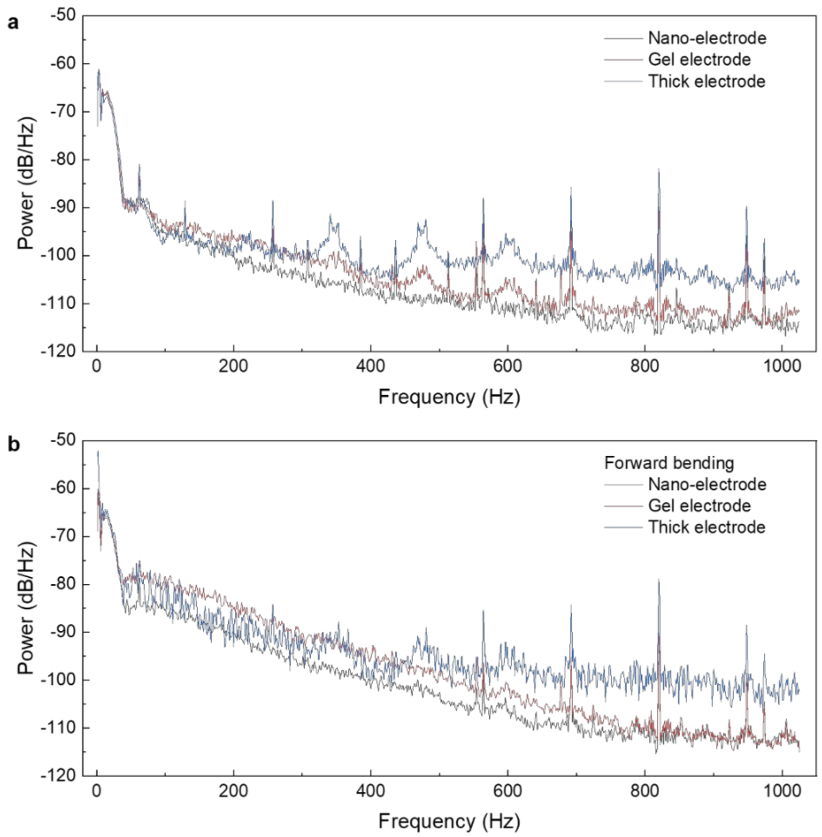

Figure S20. Power spectral density estimates of nano- (*red*), gel (*black*), and thick (*blue*) electrodes during a) static state and b) forward bending.

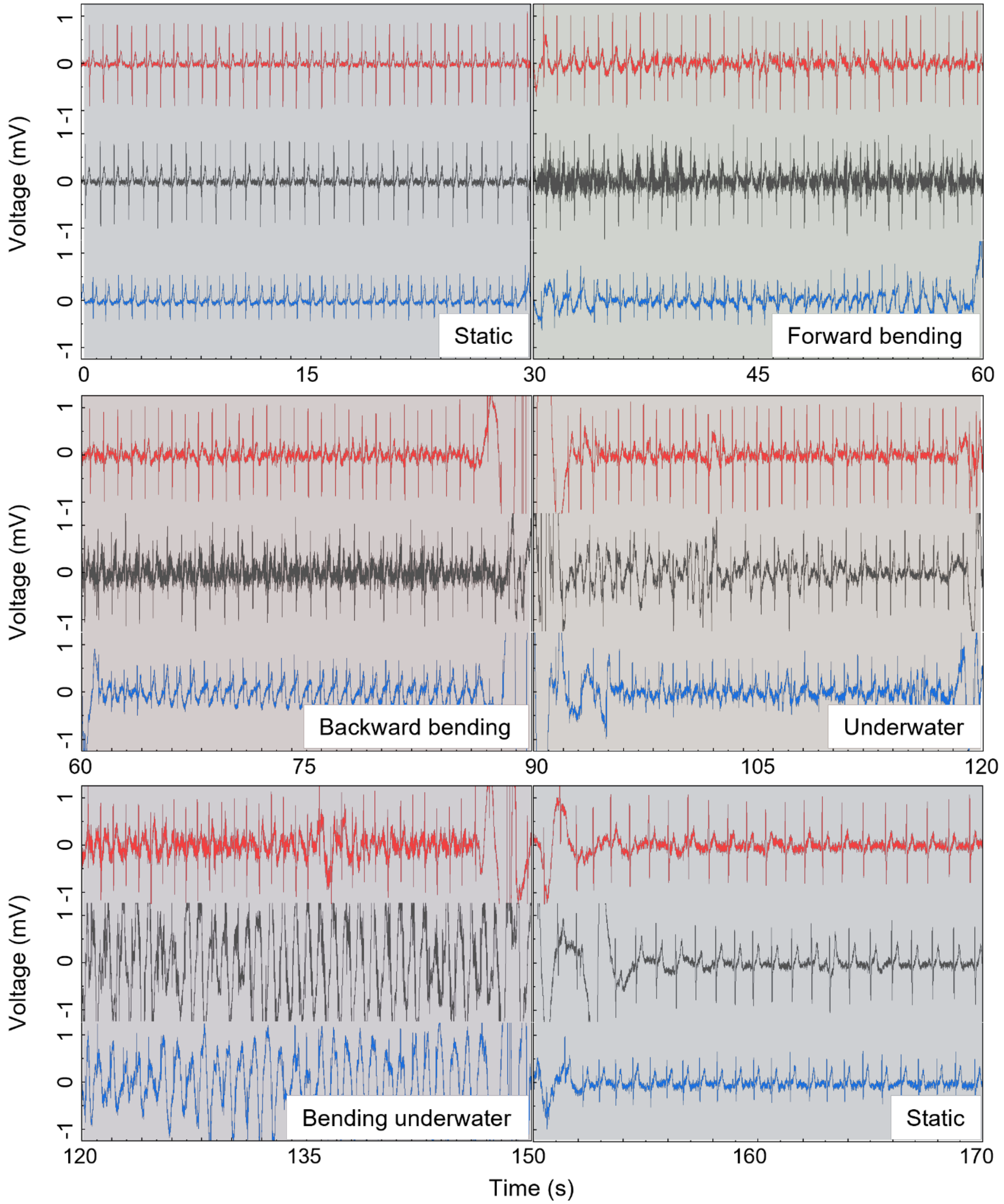

Figure S21. Continuous ECG monitoring of nano- (*red*), gel (*black*), and thick (*blue*) electrodes during activities such as at rest (*blue*), forward bending (*green*), backward bending (*red*), underwater (*orange*) and forward bending underwater (*purple*).

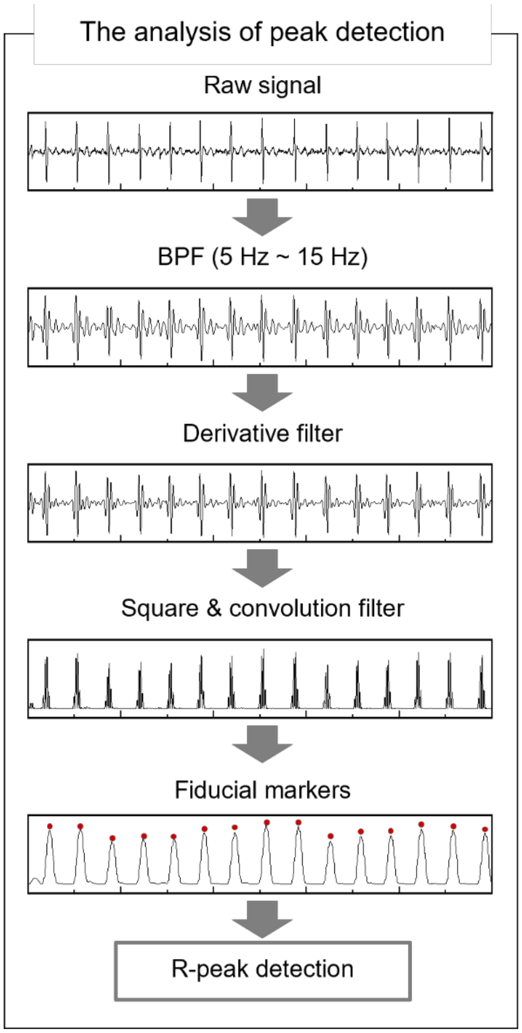

Figure S22. Block diagram of Pan-Tompkins algorithm for R-peak detection from raw ECG signals

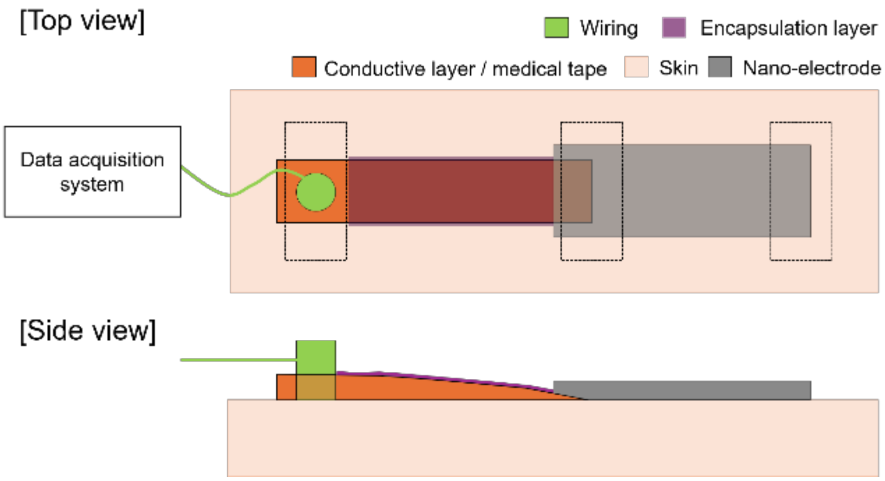

Figure S23. The schematic of interface design between the nano-electrode and the interconnected electrical lead for the human gait motion test.

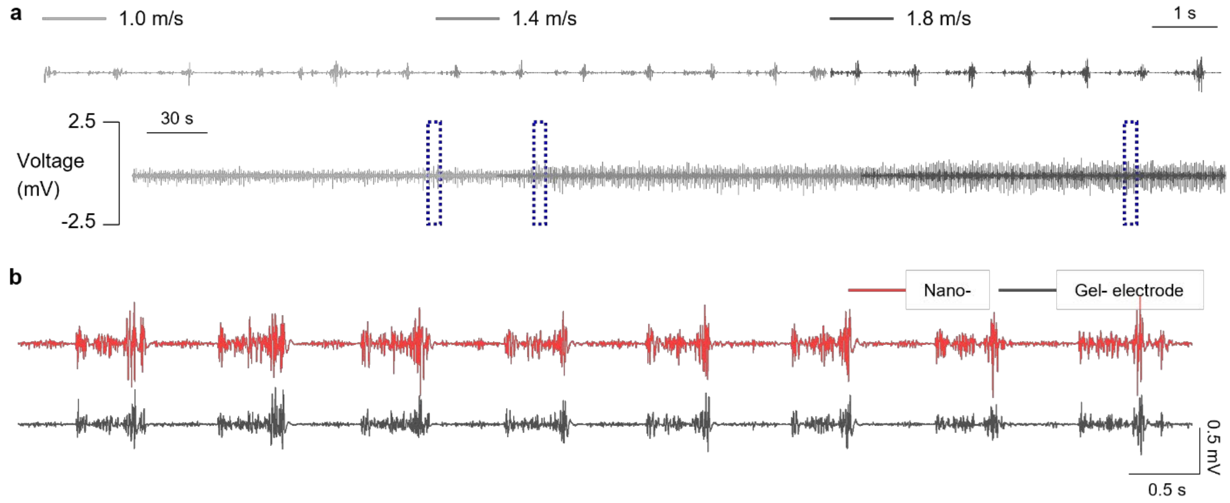

Figure S24. a) Continuous EMG monitoring of tibialis anterior muscle using gel electrodes during walking at three different speeds (1.0, 1.4, and 1.8 m/s). b) The comparison of EMG signals of nano- (red) and gel (black) electrodes in tibialis anterior muscle at 1.0 m/s.

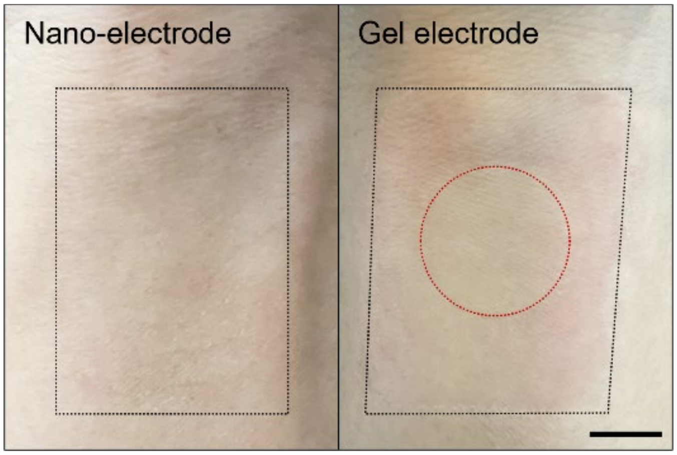

Figure S25. Photographs of before (*left*) and after (*right*) long-term use of the skin-conformal nano-electrode (*left*) and gel electrode (*right*) for 6 hours and 1 day (scale bar, 10 mm).

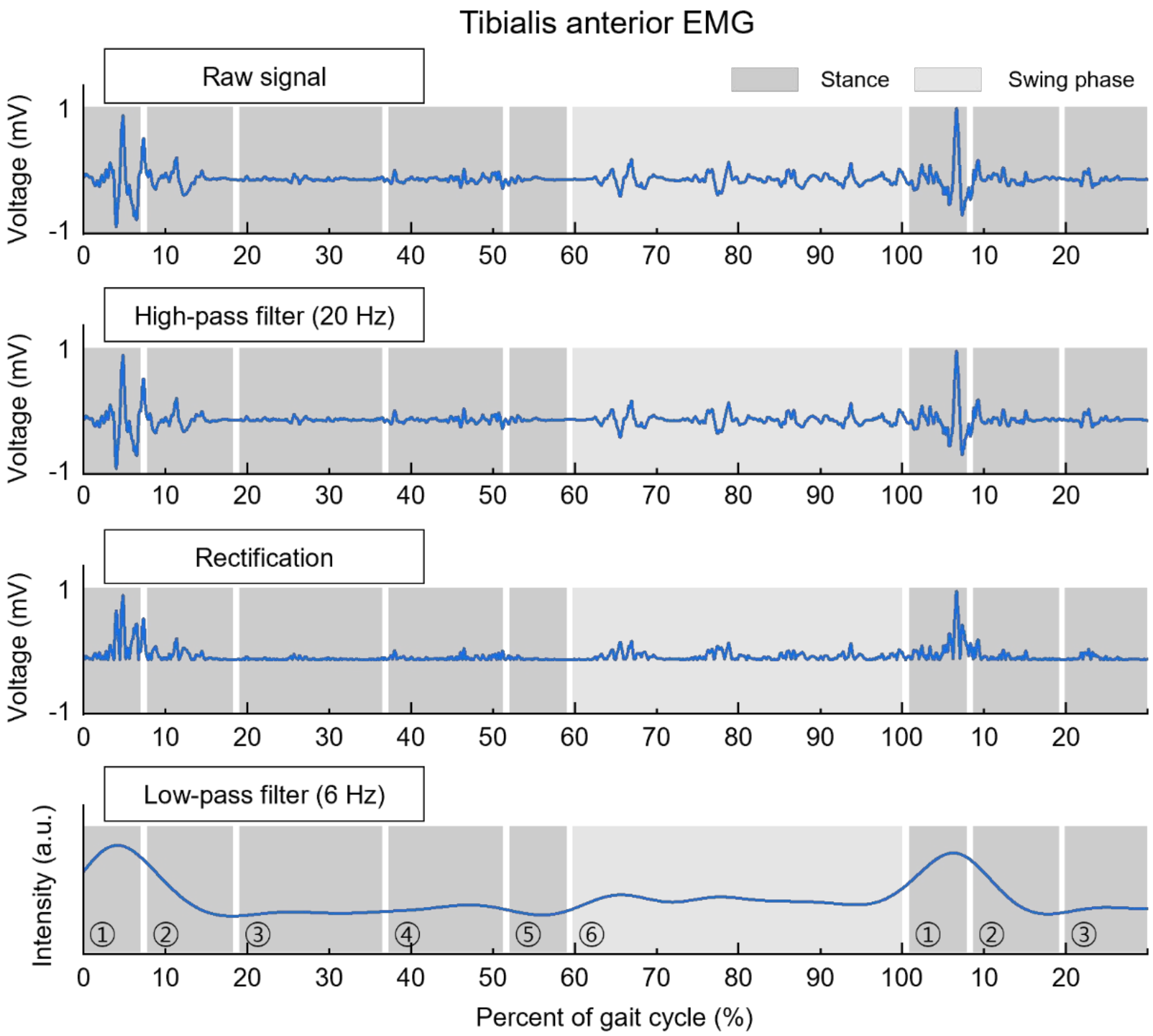

Figure S26. Signal processing protocol of the linear envelope for monitoring tibialis anterior movement. Firstly, the data is high pass filtered, followed by full wave rectification and low pass filtering to produce a linear envelope of the data.

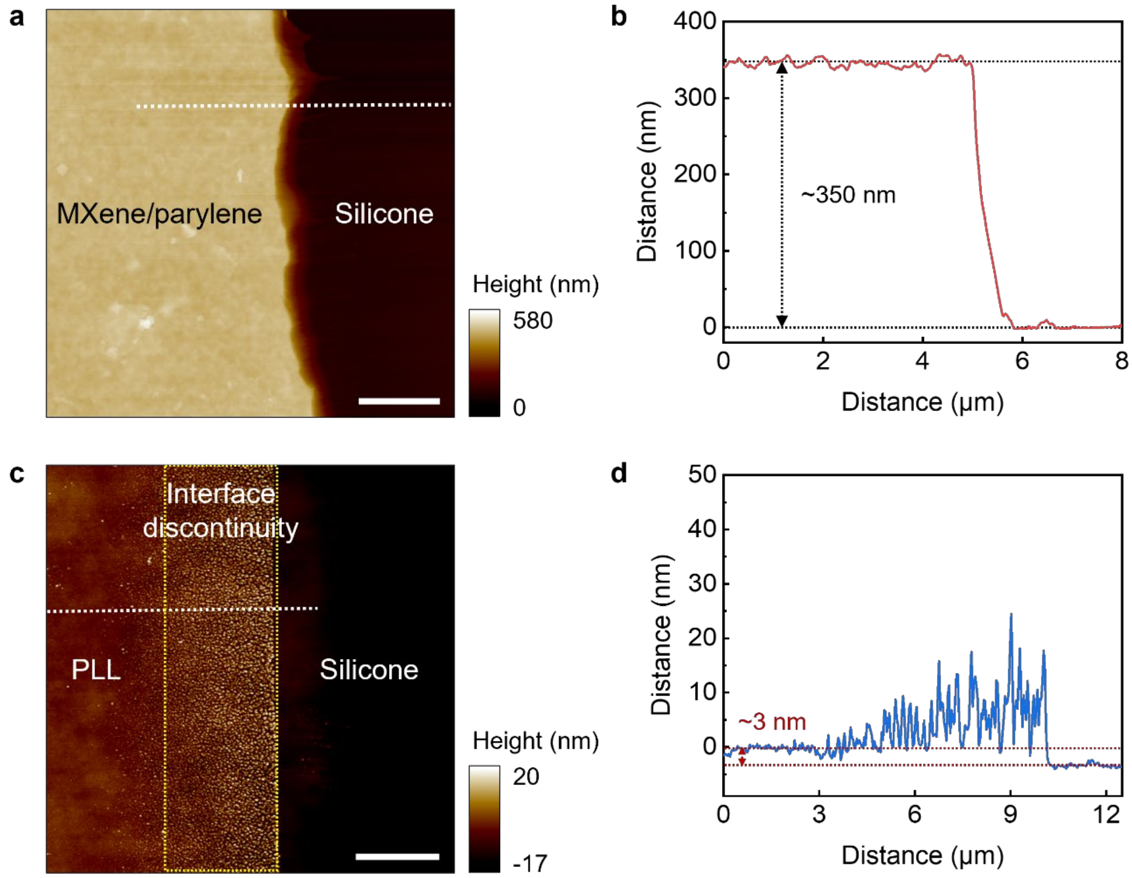

Figure S27. AFM analysis for estimating (a-b) the thickness of the skin-conformal nano-electrodes (scale bar, 2 μm) and (c-d) the PLL layer (scale bar, 4 μm).

Table S1. Summary of electrophysiological electrodes with materials, thickness and performances.

Journal	Conductor	Conductor thickness	Substrate (composite)	Substrate thickness	Encapsulation layer on electrode	Analytic study of conformal contact	SNR in ECG (gel electrode)	Signal (S) and noise (N) frequency range	Motion artifact monitoring	Underwater condition	High temperature	Long-term	Ref.
Adv. Mater. 2023	MXene/ PEDOT:PSS	20 nm	Silicon wafer Glass Tattoo paper	No info.	No	No	28 dB (25 dB)	No info.	Yes	No	No	No	[16]
Nat. Electron. 2022	Au	50 nm	PDMS	1.2 µm	Yes (PDMS)	No	37.5 dB	No info.	No	Yes	No	8 hours	[17]
Adv. Sci. 2019	Au	100 nm	PET	12.5 µm	Yes (Tegaderm)	No	16 dB	S: 2-40 Hz	Yes	No	No	No	[18]
npj. Flex. Electron. 2022	Au	17 nm	SEBS	200 nm	Yes (SEBS)	No	8 dB	S:1-100 Hz	Yes	No	No	No	[19]
Nat. Commun. 2020	Ag/ PEDOT:PSS	0.1-10 µm	Free standing	N/A	Yes (Liquid bandage)	No	48 dB (48 dB)	S: <100 Hz N: 800-900 Hz	Yes	No	No	No	[11]
Science 2022	MoS$_2$	30 nm	Free standing	N/A	No	Yes	49.8 dB (44.3 dB)	S: <100 Hz N: 800-900 Hz	Yes	No	No	No	[14]
Nat. Commun. 2020	PEDOT:PSS	-	(WPU/ D-sorbitol)	Composite (12-55 µm)	No	No	No info.	S:0.5-150 Hz	Yes	Yes	No	16 hours	[20]
Nature 2023	AuNP	45-60 nm	SEBS	2-4 µm	Yes (SEBS)	No	-	-	No	Yes	No	No	[21]
Adv. Mater. 2024	Ag@AgCl PEDOT:PSS nanowire	No info.	Ecoflex00-45 PET	No info.	No	No	26.5 dB	S: 5-50 Hz	No	Yes	No	48 hours	[22]
Nat. Commun. 2024	MXene:CNF :PCE composite	No info.	TPU-laminated cotton fabric	No info.	No	No	21 dB	S: 0.5-35 Hz	No	No	No	20 min	[23]
Adv. Mater. 2020	Au/pDAM (DMA/AA/ MEA)	Au: 80 nm pDAM: 160 µm	PDMS	70 µm	No	No	No info.	No info.	Yes	Yes	No	1 hour	[24]
Adv. Mater. 2024	PEDOT:PSS/ PVA/ d-sorbitol	370 nm	PDMS	2 µm	Yes (PDMS)	Yes	No info.	No info.	Yes	Yes	No	7 days	[25]
Sci. Adv. 2024	Gelatin/ Na$_2$SO$_4$ hydrogel	10 µm	(PU nanomesh)	Hydrogel	No	No	32.2 dB	S: 0.02-50 Hz	Yes	No	No	8 days	[26]
Adv. Funct. Mater. 2023	MXene/ WPU	No info.	TPU	No info.	Yes (TPU)	No	No info.	No info.	Yes	Yes	No	No	[27]
ACS Nano	Graphene	-	PMMA	Total ~463 nm	No	No	15.22 dB (11 dB)	No info.	Yes	No	No	No	[28]
NPG Asia Mater. 2024	SWCNT	~74 nm	SBS	356 nm	No	Yes	EMG; 24.3 ± 2.3 dB (33.3 ± 3.5 dB)	No info.	No	No	No	No	[29]
**This work**	**MXene**	**50 nm**	**Parylene**	**300 nm**	**No**	**Yes**	**35.1 dB (35.0 dB)**	**S: <100 Hz N: 100-1000 Hz**	**Yes**	**Yes**	**Yes**	**30 hours**	

Table S2. Side-by-side comparison of the proposed skin-conformal nano-electrode and the stretchable elastic conductor highlighting structural, functional, and application-level distinctions.

	Nat. Electron. **2022**, *5*, 784	**This work**
Structural design	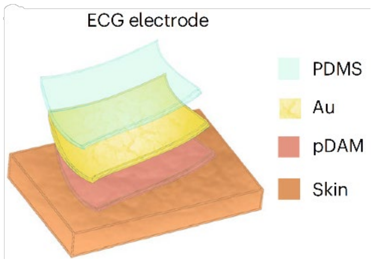	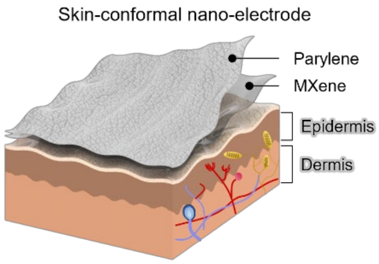
Materials	• Conductor: Au (50 nm)<br>• Substrate: PDMS (1.2 μm)<br>• Adhesion layer to skin: pDAM (22 nm)	• Conductor: MXene (50 nm)<br>• Substrate: parylene (300 nm)
Total thickness	• ~1.3 μm **(3.7 times thicker)**	• **~350 nm**
Adhesion strategy	• **Requires an additional adhesive layer** (pDAM)	• Direct skin contact **without chemical adhesives**.
Surface chemistry	• Not discussed	• hydrophilic/hydrophobic asymmetry
Signal application	• ECG (Additional layer: pDAM)<br>• *In vivo* nerve interface (Additional layer: PEDOT/CNTs)	• ECG (W/o additional layer)<br>• EMG (W/o additional layer)
Analytic study of conformal contact	• Not discussed	• Yes
Impedance analysis in underwater	• Not discussed	• Yes
Long-term stability	• 8 hours	• 30 hours

References

1) Kim, Y.; Suh, J. M.; Shin, J.; Liu, Y.; Yeon, H.; Qiao, K.; Kum, H. S.; Kim, C.; Lee, H. E.; Choi, C.; Kim, H.; Lee, D.; Lee, J.; Kang, J.-H.; Park, B.-I.; Kang, S.; Kim, J.; Kim, S.; Perozek, J. A.; Wang, K.; Park, Y.; Kishen, K.; Kong, L.; Palacios, T.; Park, J.; Park, M.-C.; Kim, H.; Lee, Y. S.; Lee, K.; Bae, S.-H.; Kong, W.; Han, J.; Kim, J. Chip-less wireless electronic skins by remote epitaxial freestanding compound semiconductors. *Science* **2022**, *377* (6608), 859–864.
2) Kwak, M. K.; Jeong, H.-E.; Suh, K. Y. Rational design and enhanced biocompatibility of a dry adhesive medical skin patch. *Adv. Mater.* **2011**, *23* (34), 3949–3953.
3) Villa, M.; Hale, R. D.; Ewing, M. Effects of fiber volume on modal response of through-thickness angle interlock textile composites. *Open J. Compos. Mater.* **2014**, *4* (1), 40–46.
4) Kim, J.; Wang, Y.; Park, H.; Park, M. C.; Moon, S. E.; Hong, S. M.; Koo, C. M.; Suh, K.-Y.; Yang, S.; Cho, H. Nonlinear frameworks for reversible and pluripotent wetting on topographic surfaces. *Adv. Mater.* **2017**, *29* (7), 1605078.
5) Kim, Y.; Mahmood, M.; Lee, Y.; Kim, N. K.; Kwon, S.; Herbert, R.; Kim, D.; Cho, H. C.; Yeo, W. All-in-one, wireless, stretchable hybrid electronics for smart, connected, and ambulatory physiological monitoring. *Adv. Sci.* **2019**, *6* (17), 1900939.
6) Lipatov, A.; Lu, H.; Alhabeb, M.; Anasori, B.; Gruverman, A.; Gogotsi, Y.; Sinitskii, A. Elastic properties of 2D Ti3C2Tx MXene monolayers and bilayers. *Sci. Adv.* **2018**, *4* (6), eaat0491.
7) Sim, W.; Kim, B.; Choi, B.; Park, J.-O. Theoretical and experimental studies on the parylene diaphragms for microdevices. *Microsyst. Technol.* **2005**, *11* (1), 11–15.
8) Ohtsuki, R.; Sakamaki, T.; Tominaga, S. Analysis of skin surface roughness by visual assessment and surface measurement. *Opt. Rev.* **2013**, *20* (2), 94–101.
9) Li, C.; Guan, G.; Reif, R.; Huang, Z.; Wang, R. K. Determining elastic properties of skin by measuring surface waves from an impulse mechanical stimulus using phase-sensitive optical coherence tomography. *J. R. Soc. Interface* **2012**, *9* (70), 831–841.
10) Gould, J. Superpowered skin. *Nature* **2018**, *563* (7732), S84–S85.
11) Ershad, F.; Thukral, A.; Yue, J.; Comeaux, P.; Lu, Y.; Shim, H.; Sim, K.; Kim, N.-I.; Rao, Z.; Guevara, R.; Contreras, L.; Pan, F.; Zhang, Y.; Guan, Y.-S.; Yang, P.; Wang, X.; Wang, P.; Wu, X.; Yu, C. Ultra-conformal drawn-on-skin electronics for multifunctional motion artifact-free sensing and point-of-care treatment. *Nat. Commun.* **2020**, *11* (1), 3823.
12) Bergey, G. E.; Squires, R. D.; Sipple, W. C. Electrocardiogram recording with pasteless electrodes. *IEEE Trans. Biomed. Eng.* **1971**, *BME-18* (3), 206–211.
13) Spach, M. S.; Barr, R. C.; Havstad, J. W.; Long, E. C. Skin-electrode impedance and its effect on recording cardiac potentials. *Circulation* **1966**, *34* (4), 649–656.
14) Yan, Z.; Xu, D.; Lin, Z.; Wang, P.; Cao, B.; Ren, H.; Song, F.; Wan, C.; Wang, L.; Zhou, J.; Zhao, X.; Chen, J.; Huang, Y.; Duan, X. Highly stretchable van der Waals thin films for adaptable and breathable electronic membranes. *Science* **2022**, *375* (6583), 852–859.
15) Jiang, Y.; Ji, S.; Sun, J.; Huang, J.; Li, Y.; Zou, G.; Salim, T.; Wang, C.; Li, W.; Jin, H.; Xu, J.; Wang, S.; Lei, T.; Yan, X.; Peh, W. Y. X.; Yen, S.-C.; Liu, Z.; Yu, M.; Zhao, H.; Lu, Z.; Li, G.; Gao, H.; Liu, Z.; Bao, Z.; Chen, X. A universal interface for plug-and-play assembly of stretchable devices. *Nature* **2023**, *614* (7948), 456–462.
16) Song, D.; Li, X.; Jang, M.; Lee, Y.; Zhai, Y.; Hu, W.; Yan, H.; Zhang, S.; Chen, L.; Lu, C. Wearable and implantable electronics: From materials to devices. *Adv. Mater.* **2023**, *35* (31), 2304956.

17) Jiang, Z.; Chen, N.; Yi, Z.; Zhong, J.; Zhang, F.; Ji, S.; Liao, R.; Wang, Y.; Li, H.; Liu, Z. A 1.3-micrometre-thick elastic conductor for seamless wireless bioelectronics. *Nat. Electron.* **2022**, *5* (11), 784–791.
18) Ha, T.; Tran, J.; Liu, S.; Jang, H.; Jeong, H.; Mitbander, R.; Huh, H.; Qiu, Y.; Duong, J.; Wang, R. L. A chest-laminated ultrathin and stretchable e-tattoo for the measurement of electrocardiogram, seismocardiogram, and cardiac time intervals. *Adv. Sci.* **2019**, *6* (14), 1900290.
19) Xie, R.; Li, Q.; Teng, L.; Cao, Z.; Han, F.; Tian, Q.; Sun, J.; Yu, M.; Zhao, Y.; Qi, D. Liquid metal-based soft electronics for smart healthcare. *npj Flex. Electron.* **2022**, *6* (1), 75.
20) Zhang, L.; Kumar, K. S.; He, H.; Cai, C. J.; He, X.; Gao, S.; Yue, S.; Li, C.; Seet, R. C.-S.; Ren, H. Fully organic compliant dry electrodes self-adhesive to skin for long-term motion-robust epidermal biopotential monitoring. *Nat. Commun.* **2020**, *11* (1), 4683.
21) Jiang, Y.; Ji, S.; Sun, J.; Huang, J.; Li, Y.; Zou, G.; Salim, T.; Wang, C.; Li, W.; Jin, H. A universal interface for plug-and-play assembly of stretchable devices. *Nature* **2023**, *614* (7948), 456–462.
22) Hu, R.; Yao, B.; Geng, Y.; Zhou, S.; Li, M.; Zhong, F.; Sun, F.; Zhao, H.; Wang, J.; Ge, J. An intrinsically adhesive and self-healing epidermal sensor derived from a hydrogel of dynamic metal-ligand interactions. *Adv. Mater.* **2024**, *36* (29), 2403111.
23) Lee, S.; Ho, D. H.; Jekal, J.; Cho, S. Y.; Choi, Y. J.; Choi, Y. Y.; Oh, S.; Lee, T.; Jang, K.-I.; Cho, J. H. A multimodal sensing device for simultaneous measurement of energy expenditure and pulse wave velocity. *Nat. Commun.* **2024**, *15* (1), 5974.
24) Ji, S.; Wan, C.; Wang, T.; Li, Q.; Chen, G.; Wang, J.; Liu, Z.; Yang, H.; Liu, X.; Chen, X. Water-resistant conformal electronics through additive gradient interfacial segregation. *Adv. Mater.* **2020**, *32* (31), 2001496.
25) Shin, J. H.; Choi, J. Y.; June, K.; Choi, H.; Kim, T.-i. An electronic skin with multimodal sensing and machine-learning-assisted health monitoring. *Adv. Mater.* **2024**, *36* (15), 2313157.
26) Zhang, Z.; Yang, J.; Wang, H.; Wang, C.; Gu, Y.; Xu, Y.; Lee, S.; Yokota, T.; Haick, H.; Someya, T. A permeable on-skin biosensing system for motion artifact correction and health indicators extraction. *Sci. Adv.* **2024**, *10* (1), eadj5389.
27) Hao, Y.; Yan, Q.; Liu, H.; He, X.; Zhang, P.; Qin, X.; Wang, R.; Sun, J.; Wang, L.; Cheng, Y. A skin-interfaced, miniaturized platform for triggered induction of skin pigmentation. *Adv. Funct. Mater.* **2023**, *33* (39), 2303881.
28) Kabiri Ameri, S.; Ho, R.; Jang, H.; Tao, L.; Wang, Y.; Wang, L.; Schnyer, D. M.; Akinwande, D.; Lu, N. Graphene electronic tattoo sensors. *ACS Nano* **2017**, *11* (8), 7634–7641.
29) Horii, T.; Yamashita, K.; Ito, M.; Okada, K.; Fujie, T. Ultrathin skin-conformable electrodes with high water vapor permeability and stretchability characteristics composed of single-walled carbon nanotube networks assembled on elastomeric films. *NPG Asia Mater.* **2024**, *16* (1), 33.
